# Modeling diadromous fish loss from historical data: Identification of anthropogenic drivers and testing of mitigation scenarios

**DOI:** 10.1371/journal.pone.0236575

**Published:** 2020-07-28

**Authors:** Marie-Line Merg, Olivier Dézerald, Karl Kreutzenberger, Samuel Demski, Yorick Reyjol, Philippe Usseglio-Polatera, Jérôme Belliard

**Affiliations:** 1 Université Paris-Saclay, INRAE, UR HYCAR, Antony, France; 2 ESE, Ecology and Ecosystems Health, INRAE, Agrocampus Ouest, Rennes, France; 3 Office Français de la Biodiversité, Direction de la Recherche, de l’Expertise et des Données, Vincennes, France; 4 UMS Patrinat (OFB-CNRS-MNHN), Muséum national d'Histoire naturelle CP41, Paris, France; 5 LIEC, CNRS UMR 7360, Campus Bridoux, Metz, France; California Academy of Sciences, UNITED STATES

## Abstract

Diadromous fishes have drastically declined over the last century, especially in Europe. Several authors have highlighted the role of large dams in this decline, but in fact, its causes are potentially multiple and cumulative, including degradation of local environmental conditions and widespread fragmentation of hydrographic networks associated with the pervasive establishment of smaller barriers. Consequently, there is a need to improve the identification and prioritization of the drivers of diadromous species loss in order to identify and apply the most appropriate conservation and restoration measures. In this study, we used both historical sources (from mid-18^th^ to early 20^th^ century) and current data to quantify the long-term loss of diadromous taxa over 555 sites throughout the French river network. Then, we modeled the effects of several anthropogenic pressures (e.g. barriers, water quality, hydrological and river morphological alterations) on diadromous taxon loss. Lastly, we assessed the potential consequences of four different scenarios of anthropogenic pressure reduction. Due to uncertainties in historical sources, some species were grouped into taxa leading to a potential underestimation of actual species extinctions. Despite this limitation, our results showed that the decline in diadromous assemblages is widespread but with contrasting magnitudes depending on site locations. The maximum height and density of barriers appeared as the major factors of taxon loss. Over the scenarios tested, we observed that exclusively improving local conditions have much more limited effects than restoring river continuity. Focusing actions on large dam removal did not show the strongest responses compared to removing medium and small-sized barriers. For effective and sustainable restoration of diadromous fish assemblage, (1) historical occurrences of diadromous fishes should be used as an indicator for assessing recovery, and (2) undertaken measures must be adapted to each basin to target and limit the number of barriers to remove while allowing diadromous fish recovery.

## Introduction

Diadromous fish species have complex life cycles which include long-distance migrations between freshwater and marine habitats to access food resources and spawning grounds. As a particular component of biodiversity [1], they constitute a remarkable ecological and conservational heritage and in some cases are recognized as keystone species of ecosystems [2]. However, due to their economic and recreational values and the numerous pressures they face through their life cycle, diadromous fishes appear to be more vulnerable to human activities than strictly freshwater fishes [3–5]. Thus, for several centuries and especially since the nineteenth century, there has been a continuous and dramatic decline in diadromous fish populations, particularly in European and North American rivers [6, 7]. As a result, many diadromous species are now classified as rare, endangered or extinct, in the IUCN Red List [8].

Most studies addressing the potential causes of declines in diadromous fish populations have cited the disruption of longitudinal continuity [7, 9, 10]. Indeed, diadromous species share a life-history that makes them particularly sensitive to river fragmentation induced by the construction of dams, weirs and sluices. Such physical barriers may reduce or compromise the accessibility of suitable habitats necessary to growth or reproduction and may, in turn, threaten the survival of species [6, 11, 12]. Moreover, the channelization of river stretches, the alteration of riparian zones, the lateral and transversal barriers and the substrate extraction have deeply impacted river morphology and sediment transport, leading to degradation of available habitats for different facets of migratory fish life cycles (e.g. spawning ground, nursery areas, shelter) [7]. Hydromorphological alterations also influence physicochemical processes (e.g. temperature, dissolved oxygen, suspended matters) and may worsen the alteration of water quality due to domestic and industrial pollution which also adversely affect diadromous species [13–15]. The causes of the decline in abundance or local extinction are varied and probably result in cumulative and/or interacting anthropogenic effects [16, 17]. However, the strong focus on the "barrier effect" induced by physical obstacles in past works may have hampered consideration of other factors. Consequently, their relative contribution to the decline in diadromous fishes has been poorly studied [18]. There is, therefore, an urgent need to develop approaches that provide reliable quantification of the specific impacts of the different anthropogenic pressures acting in streams on diadromous fish assemblage. This would help to ensure the implementation of effective mitigation measures and to provide appropriate responses to national and international regulation policies worldwide.

In the present study, we evaluated both the historical and current composition of diadromous fish assemblage throughout the metropolitan French hydrographic network, in order to identify the major factors associated with species loss in rivers and to evaluate different mitigation measures. To this end, we structured our analysis in a four-step procedure: (1) collecting historical data at the national level to reconstitute the past distribution map of diadromous taxa; (2) quantifying local taxon loss on 555 sites covering all French watercourses by comparing historical distributions with current data; (3) establishing a model linking diadromous taxon loss to several variables related to impairments in longitudinal continuity, water quality, hydrology, and river morphology; and (4) using this model for identifying and ranking the main drivers involved in the erosion of diadromous fish assemblages and for assessing the potential effects of four scenarios of human pressure reduction on the recovery of diadromous species assemblages.

## Materials and methods

### Study area

**Fig 1:**
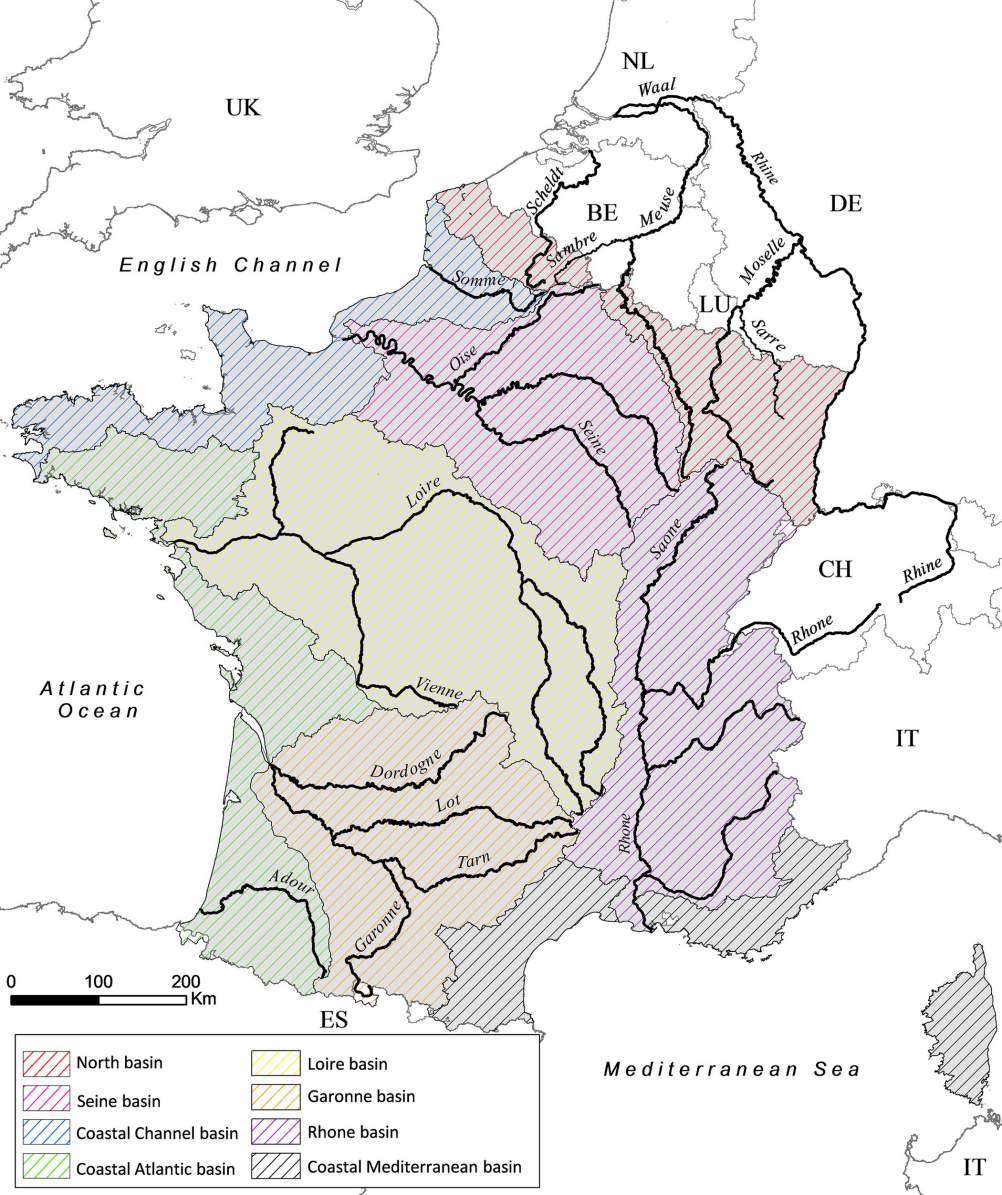
Major rivers and streams in France and large basin boundaries. BE: Belgium; DE: Germany; NL: The Netherlands; LU: Luxembourg; IT: Italy; UK: United Kingdom; ES: Spain; CH: Switzerland.

The study area includes the French metropolitan hydrographic network which represents 430,000 km, delimited by eight large basins draining 552,000 km^2^ (Fig 1). The main rivers are the Loire, Seine, Garonne and Rhone rivers whose cumulated catchments represent 62% of the metropolitan territory. The territory is geologically and climatically varied, with 22 different large hydroecoregions [19] and the majority of rivers flows in plain, with 75% of the area below 400 m elevation. The country counts 67 million inhabitants in 2019 (40 million in 1900) and the main sectors of economic activity are services followed by agriculture and industry. Nowadays, 51% of the land cover is represented by agricultural areas, 9% by urban areas (including transport and industrial areas) and 40% by natural areas.

### Diadromous fish data

#### Historical data

Historical data on diadromous fish occurrence were collected from a large range of historical sources including different kinds of public archives related to fishery and fish stocks, old scientific and naturalist literature and more occasionally iconographic sources (old photographs and postcards). In total 165 documents were consulted (S1 File).

To characterize the historical distribution of species, we focused on the period from the mid-18^th^ century to the beginning of the 20^th^ century. We selected this period because (1) it provided a lot of data on past fish distribution, unlike earlier periods, and (2) it was prior to the major transformations that affected European rivers (e.g. large dam construction, widespread industrial and domestic pollution, large scale river channelization [20]). Because some specific quantitative data (e.g. abundance, spawning grounds) was rarely available, we only considered the species occurrence.

Initially, we planned to consider all diadromous species potentially encountered in metropolitan French rivers. However, a large amount of evidence has indicated that the past distribution of eels (*Anguilla Anguilla*) was very wide; and probably due to this wide distribution, historical information for this species was too spatially imprecise to establish a suitable distribution map. Notwithstanding its interest from a conservational point of view (classified as critically endangered [21]), we excluded this species from our approach. Our work focused on 13 diadromous fish species, including one species classified as regionally extinct in the IUCN National Red List [21] and four as threatened, two of which are critically endangered (Table 1).

**Table 1:** List of the 13 diadromous species considered for taxon distribution maps; Species are associated with their national conservation status and their taxon group name if gathered.

Common name	Latin name	Taxon group name	Conservation status*
**Atlantic sturgeon**	*Acipenser oxyrinchus*	Sturgeons	RE
**European sturgeon**	*Acipenser sturio*	Sturgeons	CR
**Allis shad**	*Alosa alosa*	Shads	CR
**Twait shad**	*Alosa fallax*	Shads	NT
**Thicklip grey mullet**	*Chelon labrosus*	Mullets	DD
**Thinlip grey mullet**	*Liza ramada*	Mullets	LC
**River lamprey**	*Lampetra fluviatilis*	Lampreys	VU
**Sea lamprey**	*Petromyzon marinus*	Lampreys	EN
**Atlantic salmon**	*Salmo salar*	Diadromous salmonids	NT
**Sea trout**	*Salmo trutta*	Diadromous salmonids	LC
**Houting**	*Coregonus oxyrinchus*	-	NE
**Smelt**	*Osmerus eperlanus*	-	NT
**European flounder**	*Platichthys flesus*	-	DD

*IUCN National Red List (2019)

RE: Regionally extinct; CR: Critically endangered; EN: Endangered: VU: Vulnerable; NT: Near threatened; LC: Least concern; DD: Data deficient; NE: Not evaluated.

Due to identification uncertainties of some species in old documents (e.g. use of the common French name not precise enough to identify species), we decided to group some species under single taxon (Table 1). It should be noted that these taxa gathered species with quite similar ecological requirements. The species-level identification was kept for European flounder, houting and smelt, as no taxonomical confusion was possible for these species. Thus, eight different taxa were considered in further analyses.

Historical occurrences were then used for reconstructing species or taxon past distribution within the river network. For this purpose, we georeferenced all past occurrences (n = 1830). This task was made difficult by the lack of precision of some historical data. When an occurrence had not been precisely located in historical documents, we applied the following conservative approach: (1) when a species was assigned to a river within an administrative district (without more spatial precision), we georeferenced this occurrence to the most downstream section of the river in that district; (2) when the presence of a species was assigned to a river without any additional precision on its location, we georeferenced this occurrence on the downstream section of the river (in practice ca. 5 km from the confluence in the case of a tributary to a larger river and ca. 10 km from the sea for a river flowing directly into the sea). A historical distribution map was generated for each taxon based on occurrence data, assuming that the considered taxon was present in the whole downstream hydrographic network for each observation. The dataset of historical observations has been made available online on the GBIF [22].

#### Current data

Current data on diadromous fish occurrences were collected by The French Office of Biodiversity (OFB) over 1948 sites in France for the period 1978–2007. For each site, this dataset provides expertise on presence/absence of diadromous species, specifying natural presences or/and from fish stocking. Since we focused on sustainable natural populations, we excluded cases where the presence of a species was only attributed to restocking operations. To maintain consistency with historical data, we excluded eel data and grouped the species in the same way we did with historical data. Current occurrence data were then integrated into a Geographical Information System, and current distribution maps were generated.

### Loss of diadromous fishes

In order to assess the loss of diadromous fish at the assemblage level, we compared the current and historical lists of taxa recorded for each of the 1948 sites. Initially, we planned to base our assessment of diadromous fish loss on a ratio between the number of current and historical taxa. However, for about 11% of sites, we noticed that one or more taxa currently mentioned did not appear in historical occurrences. We assumed that these situations were essentially a consequence of gaps in knowledge of the historical distributions of species rather than true species colonization in areas they had never occupied before. We based our assumption on the fact that the vast majority of these cases concerned (1) taxa of low economic interest that consequently receiving less attention in historical sources (e.g. mullets, lampreys or to a lesser extent shads) and/or (2) small rivers of less importance that were more rarely mentioned in historical sources. To tackle this issue, we calculated the potential diadromous taxon richness corresponding to the total number of taxa identified in both historical and current records. Then, at the site scale, we based our assessment of the Loss of Diadromous Fish taxa (LDF) on a ratio between the current and potential taxon richness (adapted from a metric developed in the European EFI+ project [23]):

LDF=1–(CurrentRichness/PotentialRichness)

An LDF value of “1” corresponds to the loss of all taxa historically recorded; whereas an LDF value of “0” means that no historically recorded taxa has been lost. On a total number of 1948 sites, 1393 (71.5% of the dataset) had neither historical nor current occurrence data. The LDF variable was calculated for 555 sites (29.5% of the dataset).

In addition, for each taxon, we assessed the loss of the distribution range throughout the whole metropolitan French hydrographic network by dividing the river length currently occupied by the river length potentially occupied (combining past and current distribution ranges).

### Environmental data

Environmental variables reflecting natural conditions, fragmentation (presence of barriers), water quality and morphological and hydrological alterations were compiled for each of the 555 sites in order to study the possible links between diadromous fish loss and natural and anthropogenic river features.

The distance to the sea ("dist_sea"), the altitude ("alti") and the upstream catchment area ("area") were calculated for each site. These variables were useful to take into account the natural context of the sites and their position along the hydrographic network. Moreover, considering that species composition of diadromous fish assemblage could naturally vary according to biogeographical regions (e.g. between the Atlantic and Mediterranean basins), we defined eight hydrographic units, adapted from Oberdorff et al. [24], to integrate a possible biogeographic influence on species loss. These hydrographic units (defined under the variable "basin") identify large river basins or aggregate smaller coastal catchments according to the sea into which they flow (Fig 1).

To characterize river fragmentation downstream of each site, we used the ROE database (Référentiel des Obstacles à l'Écoulement) [25]. This database provides localization and characteristics of barriers in the French river network (e.g. dams, weirs, locks, sluices, obstacles induced by bridges) as well as information on fish pass facilities if existing. In this database, a selection was conducted to remove barriers with no expected impact on diadromous fishes. As a result, barriers listed as "in project", "in construction" and "destroyed" were not retained. Only barriers across the river course were considered. Longitudinal embankments, levees and fish farm grids were therefore excluded. To avoid bias, when several river branches existed, only the barriers located in the main stem were considered. Thereby, more than 70,000 barriers were selected. The height of barriers is relevant information to estimate the difficulty for fish to migrate in the river network [26]. So we tried to determine this height, defined in the ROE database by the difference between the upstream and downstream water surface at low water level, for each selected barrier. In the selected database, 3% of the barriers had a null height; these barriers were not included in further analysis as they were considered "destroyed". Half of the total number of barriers had a precisely defined height, while 7% of the barriers had only height range information. In that case, the mean of the range was used (for example if the height range was 1 to 2 m, the estimated height was 1.5 m). Forty percent of the barriers had no height information at all. In such a case, the height was estimated according to the barrier type and location: as applied in Miguet [27], the mean height of the same type of structure in the same hydroecoregion was allocated [19]. Given that several rivers have their downstream part outside the French territory (e.g. Rhine, Moselle, Meuse, Sambre, Scheldt), data about barriers present in Germany, Belgium, and the Netherlands were gathered from other sources. For the Rhine, Meuse, Moselle, and Sarre, data were collected from the reports of the International Commission for the Protection of the Rhine [28], the International Commission for the Meuse [29] and the International Commission for the Protection of the Moselle and Sarre [30]. These reports provide information on the main barriers (location, height and fish pass). For the Sambre, data were collected from a private website [31] and for the Scheldt from the website VisuRIS [32]. A visual inspection was performed using satellite views on Google Maps® to complete the survey and ensure an exhaustive enumeration of major barriers (e.g. dams, locks, weirs).

For each site, several variables reflecting downstream continuity (i.e. from the site to the sea) were calculated. The number of downstream barriers ("barriers") was estimated and their density ("density") was calculated by dividing the number of downstream barriers by the distance (in km) between the site and the sea [33]. The maximum height ("max_height"; i.e. the height of the highest downstream barrier) and the cumulated height ("sum_height"; i.e. the sum of the heights of all the downstream barriers) were calculated. A fish pass ratio ("pass_ratio") was calculated by dividing the number of downstream barriers with fish pass facility by the total number of downstream barriers. For the sites where no downstream barriers were recorded, the fish pass ratio was set to one. Only the fish passes of the following types were considered as relevant: pool passes, fish lifts, fish locks, counterflow passes, vertical slot passes and bypass channels [34].

Morphological and hydrological conditions of each site were characterized at the local scale. For each site, data from expert judgment had been collected for nine morphological and four hydrological alteration parameters (see [35, 36] for previous uses of these data). For each parameter, the alteration intensity was described by four levels: "Null", "Low", "Medium" and "High". We summarized morphological and hydrological alterations using multiple correspondence analyses (MCA; [37]) performed on each group of parameters. The site coordinates on the first axis of both MCA were used to create two synthetic quantitative variables ("alter_morpho" and "alter_hydro"), describing a gradient of morphological and hydrological alteration (S2 and S3 Files). The more positive the variable value was, the more altered the site was.

To characterize water quality at the site scale, we used data extracted from the online database Naïades [38], which provided quantitative information on 212 physicochemical parameters (due to their poor availability, micropollutants were excluded). For each parameter, the values were averaged over 30 years, from 1978 to 2007, corresponding to the fish expertise period considered here. These parameters were then pooled into five groups: organic, phosphorous and nitrogenous matters (excluding nitrates), nitrates, and suspended matter. For each group, the water quality status is defined by five classes: "Bad", "Poor", "Moderate", "Good", "High". The class limits were defined according to the thresholds recommended in the SEQ-Eau version 2 [39]. An MCA was used to create a synthetic variable ("alter_wq") describing a gradient of water quality alteration, using the site coordinates on the first axis (S4 File).

### Model

To analyze variations in LDF (expressed as a proportion) against environmental variables we used a logistic generalized linear mixed modeling approach (GLMM) with a binomial distribution and a logit link. Depending on their distribution, the environmental variables were transformed when necessary to avoid asymmetric distribution due to the presence of extreme values (Table 2). To ensure the relative independence of predictors and to avoid multicollinearity, we first calculated Spearman rank correlation coefficients among environmental variables. When variables were highly correlated (Spearman rho < -0.7 or > 0.7), only one of them was included in the multivariate analysis [40]. For example, the variable "dist_sea" was strongly correlated to "area", "barriers" and "alti", and the variable "max_height" was strongly correlated to "sum_height". The variables "dist_sea" and "max_height" were retained as they provided a better performance of the model (lower Akaike Information Criterion; AIC; [41]) in a preliminary analysis. The variable "density", "alter_wq", "alter_morpho", "alter_hydro" and "pass_ratio" were all selected as they were not strongly correlated to other variables. Because of the location of study sites, spatial autocorrelation might affect our analysis. To downplay the effect of spatial autocorrelation, the variable "basin" was used as a random effect factor to take into account large-scale biogeographic dissimilarities [42–45].

**Table 2:** Candidate variables to model the potential effects of environmental constraints on richness loss of diadromous fishes.

Variable	Description (units)	Transformation	Candidate for the GLMM (X)	Scale
alter_hydro	Alteration of natural hydrology (-)	-	X	Site
alter_morpho	Alteration of river morphology (-)	-	X	Site
alter_wq	Alteration of water quality (-)	-	X	Site
alti	Altitude of the site (m)	sqrt(x)		Site
area	Upstream catchment area (km^2^)	sqrt(x)		Upstream
barriers	Number of downstream barriers (-)	log(x+1)		Downstream
basin	Basin name (-)	-	X	Basin
sum_height	Cumulative heights of downstream barriers (m)	log(x+1)		Downstream
density	Density of downstream barriers (barriers/km)	sqrt(x)	X	Downstream
dist_sea	Distance between the site and the sea (km)	sqrt(x)	X	Downstream
max_height	Maximum height of downstream barriers (m)	log(x+1)	X	Downstream
pass_ratio	Ratio of downstream fish passes (-)	sqrt(arcsin(x))	X	Downstream

To identify the most relevant model we performed a multi-model selection approach [46] using the “MuMIn” package [47]. We first ranked the candidate models (covering all the possible combinations of predictor variables) according to their AICc (correct AIC for small sample size). We then identified the best models as those with the lowest AICc values and with a threshold value of ΔAICc < 2. In a next step, considering this set of best models, we applied a model averaging procedure to assess the relative importance of each predictor variable (by summing the Akaike weights of all the models that include that variable), we considered a predictor as noticeably important when its relative importance value exceeded 0.5 [48]. Finally, we built our final model including only the predictor variables identified as important in the previous step (importance value > 0.5). The variance inflation factor (VIF) was applied to detect a problem of multicollinearity [49]. Since environmental data were not available for every site, the model was built on a reduced number of sites (n = 361, 18.5% of the original dataset). Marginal and conditional R-squared were calculated to assess the variance explained by fixed and random effects [50]. To assess the predictive performance of the model, cross-validation was performed by splitting the calibration dataset into two subsets, the “training” and “test” datasets, containing respectively 70% and 30% of the calibration sites [51]. The splitting was based on a random selection but we forced the process to comply with the initial proportion of sites per basin in both datasets. The model coefficients were re-estimated using the training dataset. The model goodness of fit was evaluated both on training and test datasets using three metrics: the mean absolute error (MAE), the root mean squared error (RMSE) and the squared correlation between the response variable and the predicted values (R^2^). This operation was repeated 200 times.

The significance of the fixed term coefficients in the model was assessed with a Wald test and profiled confidence intervals were calculated. This step allowed us to precise the uncertainty around the significance of coefficients in the relationship between environmental variables and the LDF variable. To visualize the effect of environmental variables on the LDF variable, the specific marginal effect of each environmental factor was represented along the environmental gradient. The values of the other environmental variables were held constant at their median [52]. Finally, the magnitude of the random effect was assessed by comparing the basin intercepts.

### Projections under mitigation scenarios

From the model previously built, we tested the effect of four mitigation scenarios on potential diadromous fish recovery: (1) removal of all large-size barriers (height > 10 m), (2) removal of all medium-size barriers (height between 2 and 10 m), (3) removal of all small-size barriers (height < 2 m) and (4) general reduction in local alterations on river morphology, hydrology and water quality. We deliberately chose contrasting scenarios to see if very different and radical mitigation measures led to contrasted changes in diadromous loss and if these changes varied among basins. Plus, we did not take into account the possible consequences of dam removal on river morphology, hydrology and water quality. This constitutes a simplification that must be taken into account when interpreting results. In the scenario 4, hydrological and morphological variables were set to a "low" level or held to a "null" level and water quality variables set to a "good" quality level or held to a "high" quality level. The three synthetic variables were then recalculated by considering this new configuration of sites as supplementary individuals in the respective MCAs initially built. For each site, we predicted the new proportion of richness loss (predicted LDF) under each scenario and we calculated the difference in predicted LDF compared to the initial prediction (current environmental conditions). The greater the difference among the predicted LDF values, the more likely the scenario is to reduce the proportion of richness loss. Finally, for each scenario, the reduction in LDF was tested by a Fisher's exact test [53] at the basin-level and France-level. All statistical analyses and mapping were performed with R 3.4.2 [54].

## Results

### Evolution in the diadromous fish richness

**Fig 2:**
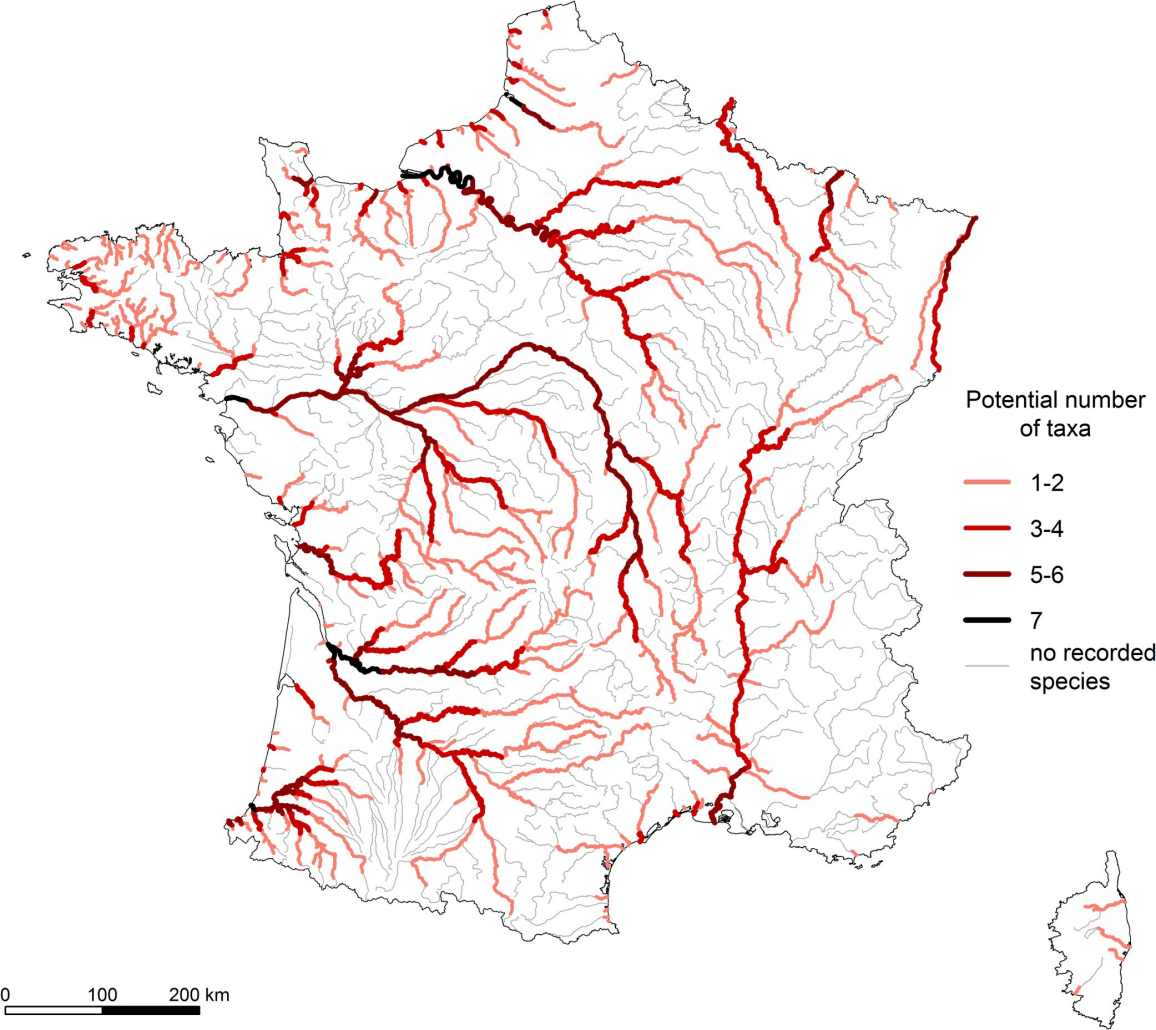
Potential richness of diadromous fishes in France. The potential number of taxa was calculated considering historical and current data. River segments without any known historical or current data appear as "no recorded species" segments.

The potential richness in diadromous taxa was high in the largest rivers (Fig 2) with a maximum of seven taxa for the Atlantic and the English Channel river systems (Seine, Loire, Adour, Dordogne, Garonne and Somme rivers–see Fig 1 for river locations). This richness was lower for the Rhone and the French part of the Rhine rivers (six taxa) and most of the rivers in the Moselle and Meuse basins (five and three taxa, respectively). Conversely, the smallest coastal basins hosted much poorer diadromous assemblage (less than four taxa with a few exceptions). Given the life history of diadromous fish species and their way of colonizing river networks, the potential richness exhibited a downstream-upstream gradient with a maximum richness in river segments close to the sea.

**Fig 3:**
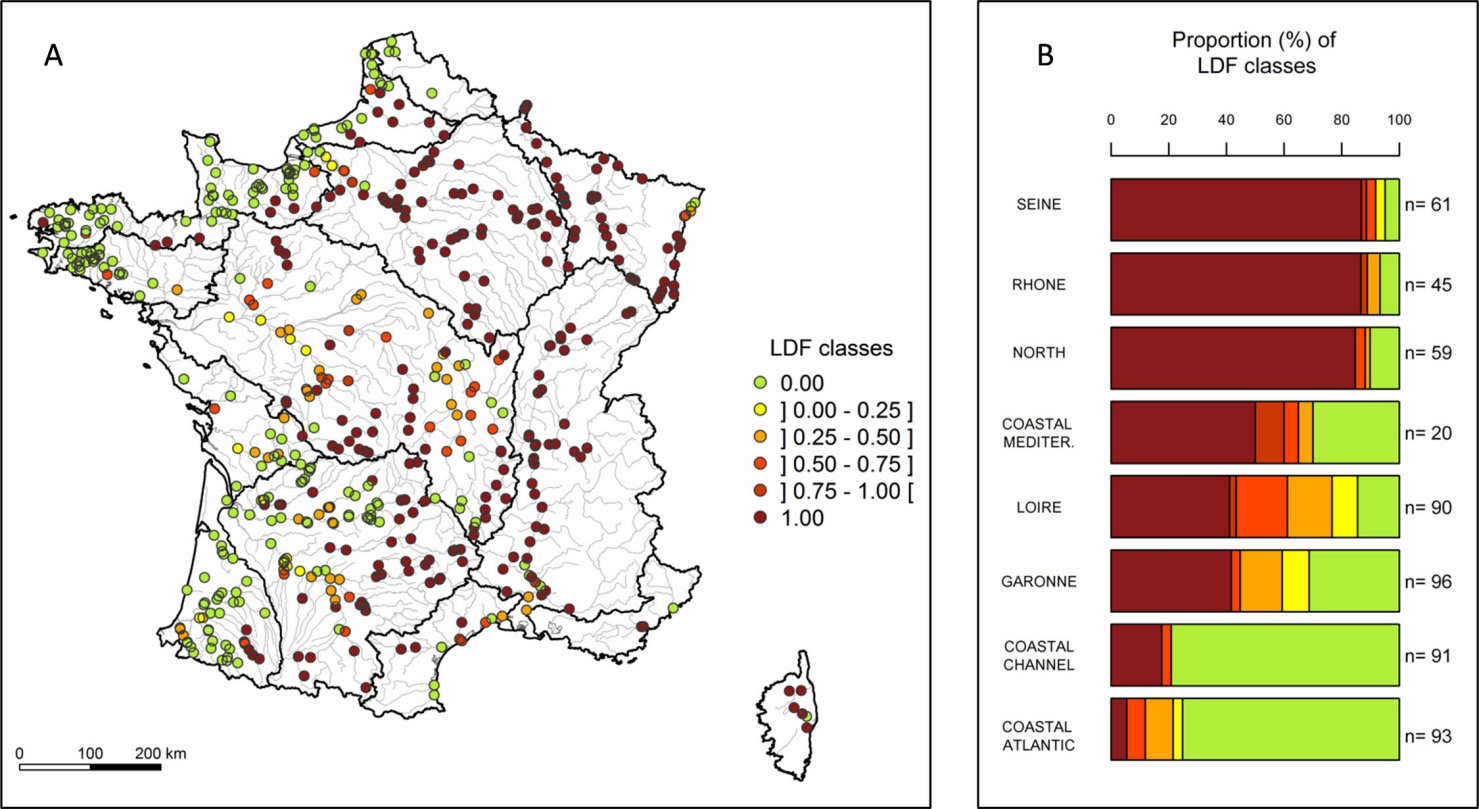
LDF classes per site (A) and proportions (%) of classes per basin (B). ‘n’ corresponds to the number of sites considered in each basin.

The loss of taxa showed considerable variation across sites (from 0 to 100%) and appeared geographically contrasted over the country (Fig 3A). The Chi2 independence test indicated a significant difference in the distribution of sites in the classes of diadromous taxon loss according to the basins (chi2 = 344; df = 35; p-value < 0.001). The Seine, Rhone and North basins experienced the most dramatic decline, with about 90% of their sites exhibiting a total loss of diadromous taxa (Fig 3B). In the North Basin, the French parts of the Meuse and the Moselle rivers lost all their diadromous taxa, three to five taxa respectively on their lower reaches (Fig 2). Similarly, a large part of the upper Seine basin showed a total loss (up to six taxa) while the downstream part partially maintained its historical richness. Three taxa remained on the downstream part of the French Rhine, while the whole historical community disappeared from its upper part. The coastal Mediterranean basin, Loire and Garonne basins seemed more heterogeneously impacted, with about 40% of their sites exhibiting a total loss of diadromous taxa but 20 to 30% of the sites showing no loss. The sites with a total loss of diadromous taxa were mainly located on the upper part of the Loire, Dordogne, Vienne, Lot and Tarn rivers. In the Mediterranean basin, the sites without any diadromous taxa remaining were rather located in the lower part of some coastal rivers. On the other hand, the coastal Atlantic and coastal Channel basins were the less impacted basins, with only 5% and 18% of sites showing a total loss of diadromous taxa, respectively. Most of the sites (ca. 80%) in these basins have indeed maintained their historical diadromous richness.

**Fig 4:**
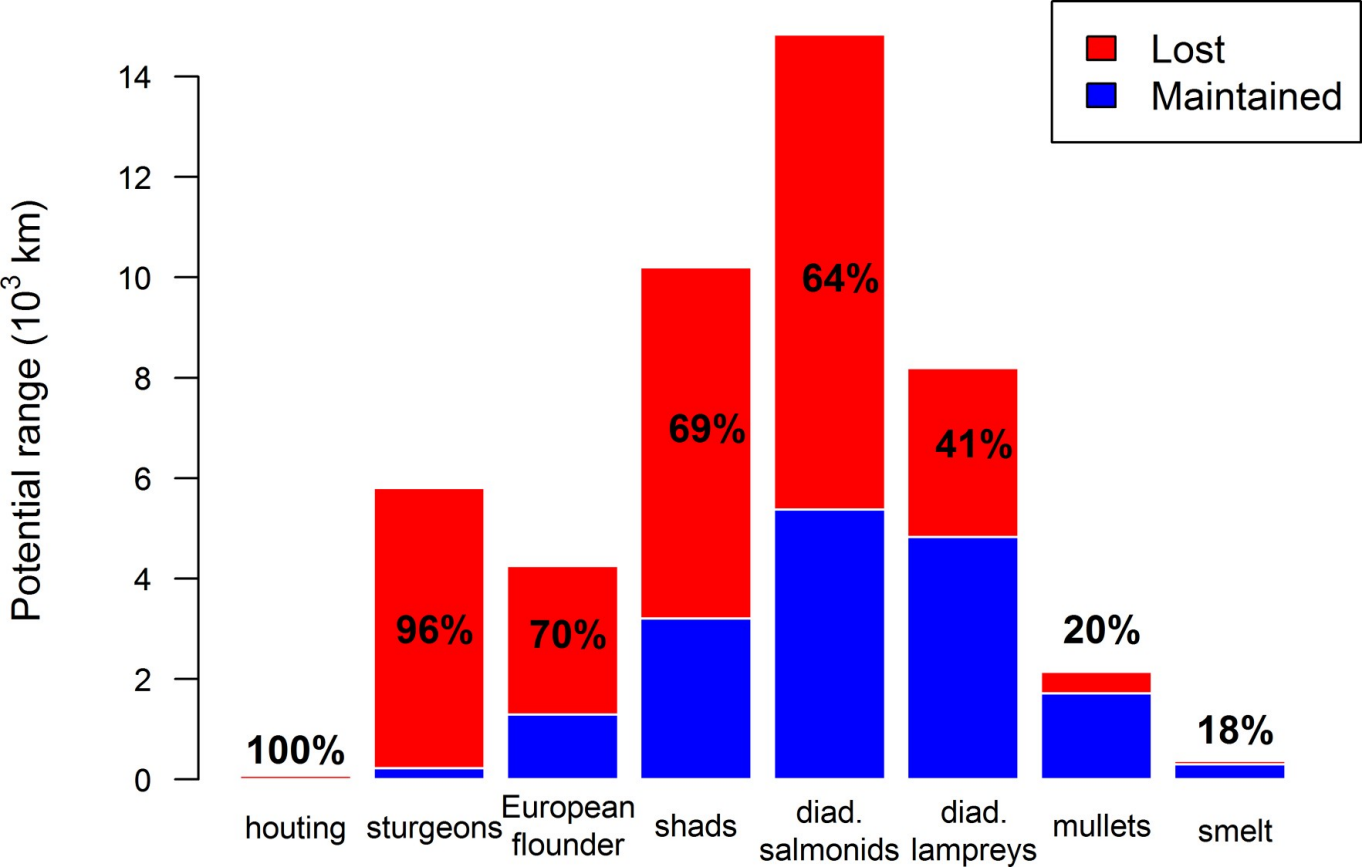
Maintained and lost potential distribution ranges of the French river system per taxon. The potential distribution range is represented in thousands of kilometers. The proportions of lost river length and maintained river length are represented in red and blue respectively and the percentage of lost length is displayed for each taxon.

The loss of distribution range within the French river network varied greatly among taxa (Fig 4). While the observations of houting were historically mentioned on the Rhine River and its distribution represented less than 100 km, the species is nowadays absent (see S5 File for potential and current distribution maps). The sturgeons, which were historically distributed in all the main French rivers (ca. 6,000 km occupied), lost 96% of their former distribution range and only a few individuals remain in the Gironde estuary, Garonne and Dordogne. The distributions of the European flounder, shads and salmonids have also dramatically declined, with a loss of 70%, 69% and 64% of their potential distribution range. This loss was especially high for salmonids and shads with respectively 9500 km and 7000 km of river length lost. Lampreys have remained broadly distributed over the Loire and Adour-Garonne basins while their distribution dropped considerably in the Seine, Rhine and Rhone basins, leading to a loss of 41% of their potential distribution range. The lowest losses concern the mullets and the smelt with a reduction of 20% and 18% of their potential distribution range, representing respectively less than 500 km and 100 km of river length.

### Main drivers of loss

Among all possible initial candidate models, six models were selected in our multi-model selection approach based on lower AICc values (see S6 File). Each of the seven environmental candidate variables was retained at least once in the selected models. However, the relative importance value of the ratio of fish passes (pass_ratio) was low (< 0.5—see S6 File); this variable was consequently removed from our final LDF model (Table 3). Despite non-significant associated p-values (above the 5% level), water quality alteration (alter_wq) and river morphology alteration (alter_morpho) were incorporated into the model (relative importance value of 0.79 and 0.55, respectively), suggesting that these variables contributed to improving the prediction of diadromous fish loss (Table 3). Multicollinearity was not detected in our final variable set (VIF < 2).

**Table 3:** Results of the generalized linear mixed model predicting the proportion of richness loss for diadromous fish.

Fixed effects	β	95% CI	p-value	Random effects	Intercept
Intercept	-7.37	[-9.97;-4.77]		Coastal Mediter.	3.28
max_height	3.76	[2.31;5.21]	<0.001	Seine	2.39
dist_sea	0.22	[0.12;0.31]	<0.001	Rhone	0.42
density	5.36	[2.02;8.69]	0.002	Coastal Channel	0.39
alter_hydro	0.65	[0.17;1.13]	0.007	North	-0.78
alter_wq	0.56	[-0.08;1.21]	0.085	Garonne	-0.86
alter_morpho	0.38	[-0.19;0.95]	0.193	Loire	-1.15
				Coastal Atlantic	-1.87

For the fixed effects: *β* coefficients of the GLMM and their 95% confidence interval (95% CI) are provided in square brackets. The significance of the fixed terms is tested with a Wald test (p-values provided). For the random effects: Intercept coefficients are provided for each basin.

Eighty percent of the overall variance was explained by both fixed and random effects (53% for fixed effects only).

The cross-validation displayed a reasonable consistency in the model performance statistics when predictions were computed (1) on the training datasets (70% of the randomly selected calibration sites) or (2) on the validation datasets (30% of the randomly selected calibration sites) with the new model coefficients established from training datasets (S7 File). This suggested that the error rates associated with new predictions on independent datasets should increase quite slightly compared to the error rates observed for the calibration dataset.

**Fig 5:**
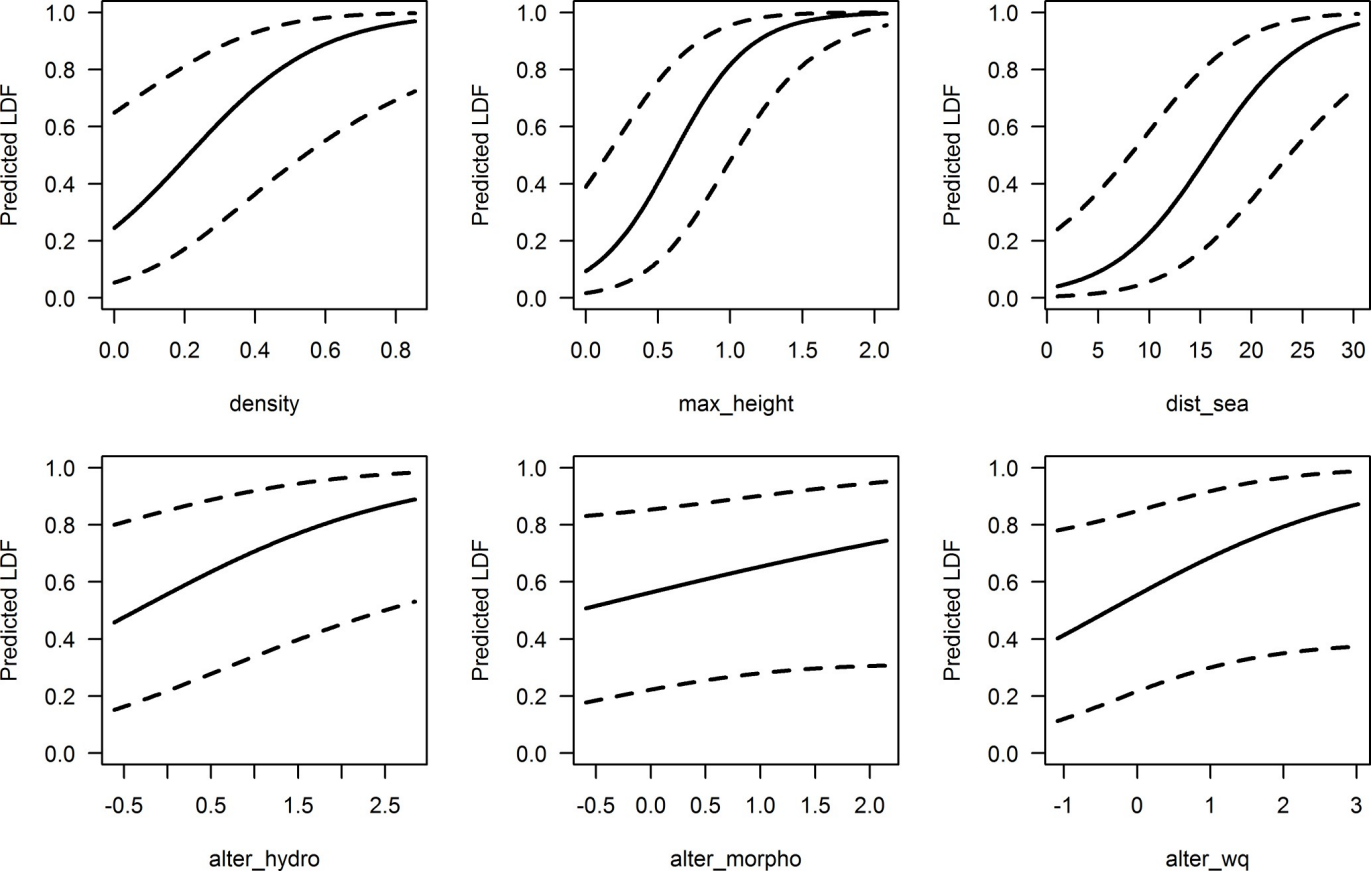
Specific marginal effect graphs from the LDF model. The graphs display the predicted LDF values (y-axis) along a gradient of a given environmental variable (x-axis). The x-axis values are transformed as described in Table 2. Lower and upper limits of the 95% confidence interval are represented by dashed lines.

The different environmental variables included in the model exhibited more or less strong links with the loss of diadromous taxa (Table 3 and Fig 5). The predicted LDF increased strongly with the distance to the sea (dist_sea), suggesting a higher vulnerability of diadromous assemblage in the most distant sites from the sea. Considering all other variables fixed at their median value, a site located 100 km (10; after transformation [Fig 5]) away from the sea had a predicted LDF of 0.2 whereas it reached nearly 0.8 at sites located 400 km away (20; after transformation). Both variables related to the presence of barriers were closely related to the loss of diadromous species. The maximum height of downstream barriers (max_height) was the variable that showed the most pronounced effect. In a median situation, a two-meters-high barrier (0.5; after transformation) resulted in a predicted LDF of 0.4. It reached 0.8 for a ten-meters-high barrier (1.0; after transformation). The rise in barrier density also strengthened the risk of diadromous species loss. This increase seemed roughly linear up to 20 barriers per 100 km (0.45; after transformation), beyond which the increasing trend slowed down. The three variables accounting for local anthropogenic alterations (hydrological regime [alter_hydro], river morphology [alter_morpho] and water quality [alter_wq]) displayed a positive link with the risk in diadromous taxon loss. Both the alteration of water quality and the alteration of river morphology did not show a significant effect (p-value > 0.05). The standard deviation of the random intercept (SD = 2.05) indicated a significant effect of the basins on LDF, suggesting inter-basin differences in LDF. The coastal Mediterranean basin and the Seine basin showed the most positive intercept, while the Coastal Atlantic and Loire basins had the most negative intercept and the Coastal Channel and Rhone basins had an intercept close to 0.

### Scenarios of reducing anthropogenic pressures

In the initial dataset (no scenario), 22 barriers were recorded downstream to each site on average with a mean density of 10 barriers/100 km (Table 4). These statistics remained nearly unchanged in the scenario 1 but were reduced in the scenario 2 (barriers_mean2_ = 16 barriers; density_mean2_ = 8 barriers/100 km) and drastically reduced in the scenario 3 (barriers_mean3_ = 6 barriers; density_mean3_ = 2 barriers/100 km). While the scenario 2 and 3 had no effect on the mean maximum height of downstream barriers, the scenario 1 reduced significantly the value of this metric (max_height_mean1_ = 4 m) compared to the initial dataset (max_height_mean0_ = 8 m). The scenario 4 resulted in a significant decrease in the mean value of the three synthetic variables of hydrological, morphological and water quality alterations regarding the initial dataset (resp. -0.2 vs 0.2; 0.001 vs 0.23 and -0.07 vs 0.14).

**Table 4:** Mean values of anthropogenic pressure variables and their standard deviations calculated on the 361 sites for the different scenarios.

	Mitigation measures	barriers	Density (barriers/100 km)	max_ height (m)	alter_ hydro	alter_ morpho	alter_ wq
**No scenario**	-	22 (22)	10 (10)	7.97 (14.18)	0.26 (0.81)	0.21 (0.74)	0.14 (0.59)
**Scenario 1**	Removal of all large-size barriers (height > 10 m)	21 (22)	9 (10)	4.03 (2.84)	0.26 (0.81)	0.21 (0.74)	0.14 (0.59)
**Scenario 2**	Removal of all medium-size barriers (height between 2 and 10 m)	16 (18)	8 (9)	6.36 (14.54)	0.26 (0.81)	0.21 (0.74)	0.14 (0.59)
**Scenario 3**	Removal of all small-size barriers (height < 2 m)	6 (8)	2 (2)	7.64 (14.34)	0.26 (0.81)	0.21 (0.74)	0.14 (0.59)
**Scenario 4**	Reduction in local alterations on river morphology, hydrology, and water quality	22 (22)	10 (10)	7.97 (14.18)	0 (0.51)	-0.19 (0.29)	-0.07 (0.37)

**Fig 6:**
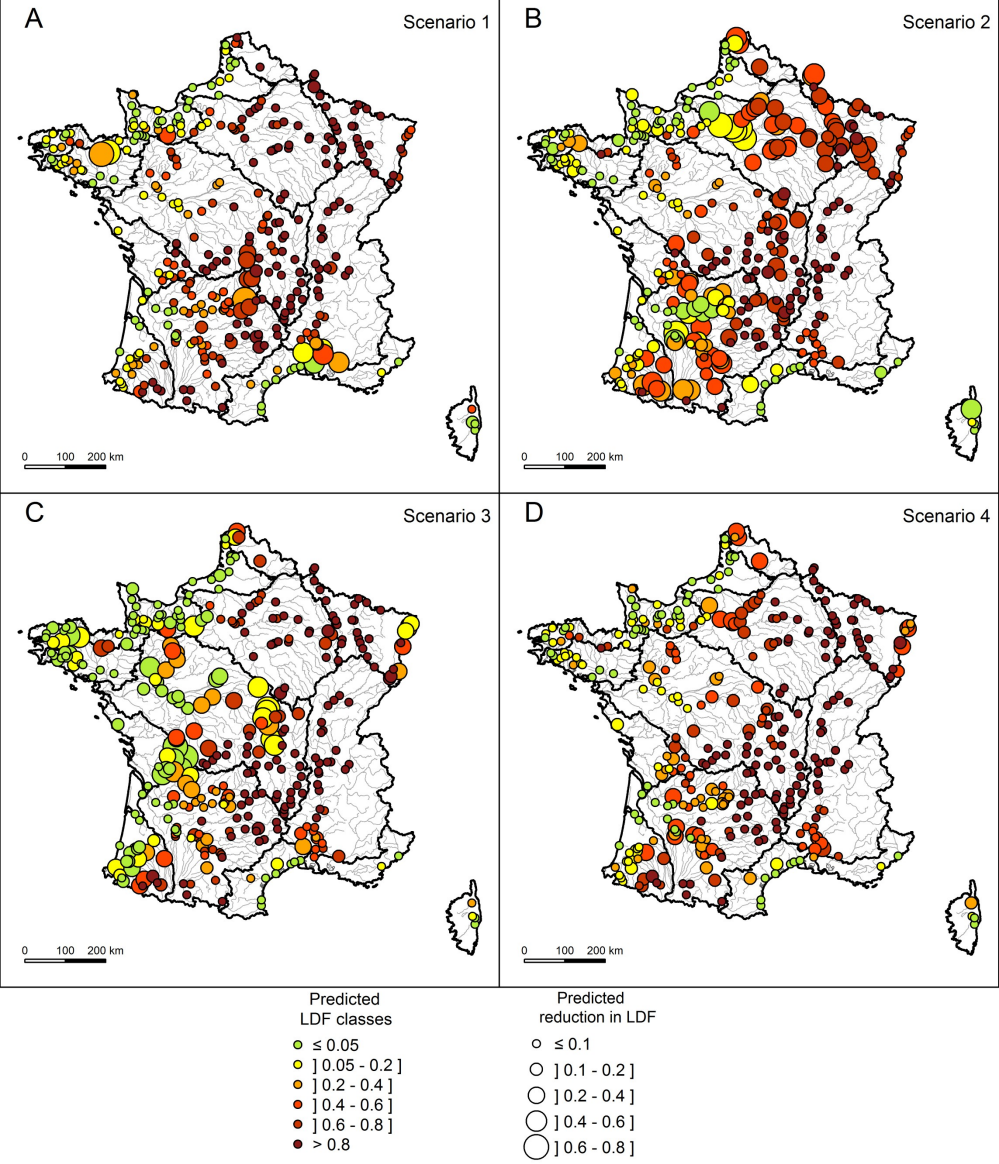
Reduction in predicted LDF induced by each scenario. The colors correspond to the LDF classes calculated (A) after removing barriers over 10 m in height (Scenario 1), (B) after removing barriers between 2 m and 10 m in height (Scenario 2), (C) after removing barriers under 2 m in height (Scenario 3) and (D) after reducing local hydrological, morphological and water quality alterations (Scenario 4). The circle size represents the predicted reduction in LDF compared to the initial conditions without any scenario.

**Fig 7:**
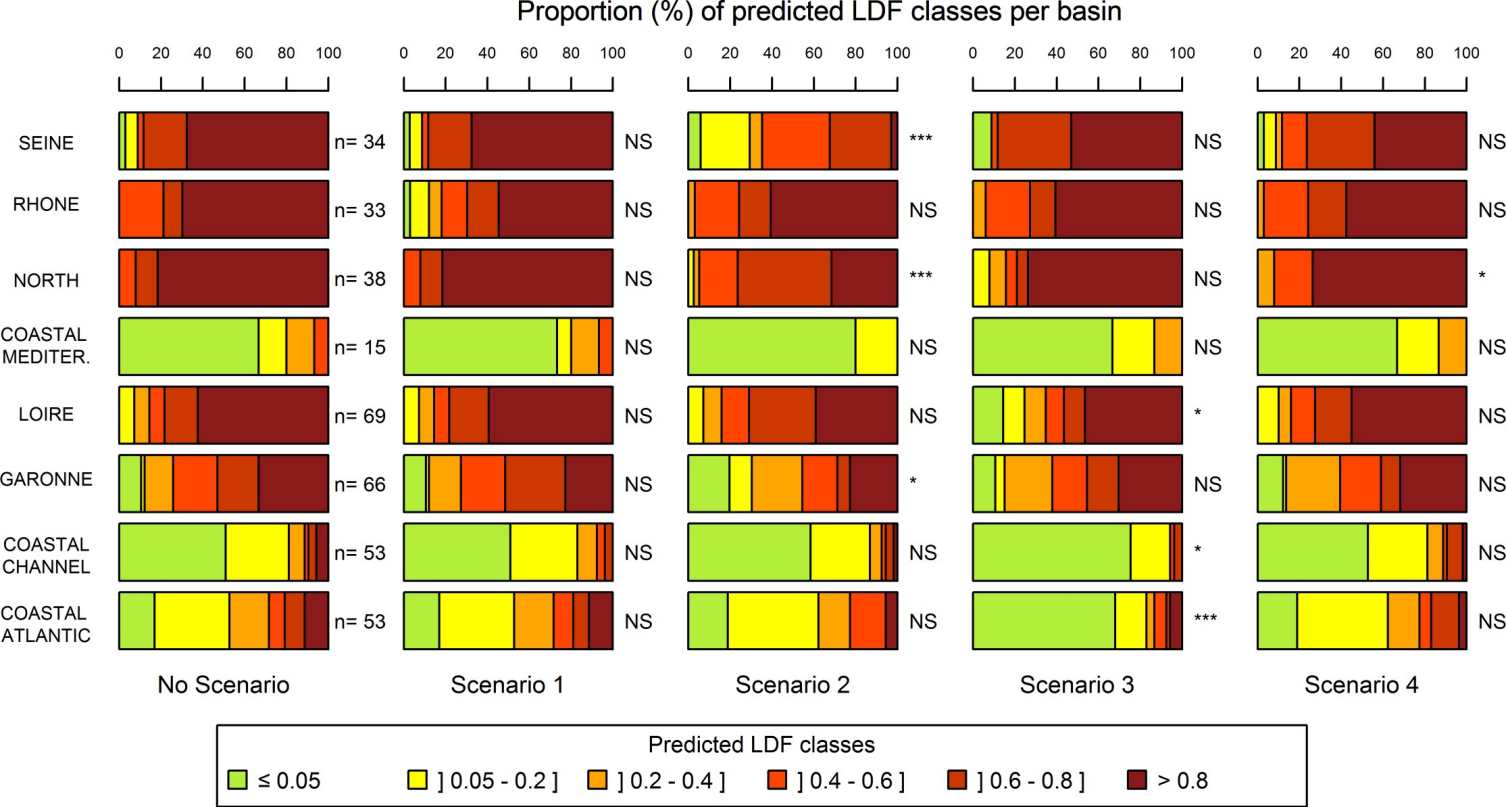
Proportions of LDF classes per basin for each scenario. The colors correspond to the LDF classes calculated without any scenario (No Scenario), after removing barriers over 10 m in height (Scenario 1), after removing barriers between 2 m and 10 m in height (Scenario 2), after removing barriers under 2 m in height (Scenario 3) and after reducing local hydrological, morphological and water quality alterations (Scenario 4). P-values significant at a 5% level (*), 1% (**), 0.1% (***) and not significant (NS) are provided. ‘n’ corresponds to the number of sites in each basin.

In comparison with the initial conditions, the predicted LDF values remained unchanged for most sites under the scenario 1 (Figs 6A and 7). Only a few sites located in the downstream part of the Rhone basin, in the central part of France and in the coastal Channel basin exhibited a decrease of LDF. But these punctual modifications were not significant for any basin. The scenario 2 showed a much more widespread reduction in the risk of loss of diadromous taxa (Figs 6B and 7). The resulting changes were particularly significant in the Seine, North and Garonne basins. In the Seine basin, the proportion of sites with a low taxon loss risk (predicted LDF < 0.2) increased from 10% to 30%, with the most important changes in the lower reaches. Whereas the taxon loss risk remained quite high in most of the North basin (predicted LDF > 0.4), the proportion of sites with very high taxon loss risk (predicted LDF > 0.8) was reduced by more than half. The scenario 3 led to a significant drop in predicted LDF in the coastal Atlantic basin with 68% of the sites having a low predicted LDF (≤ 0.05) against 17% in the initial conditions (Figs 6C and 7). In the coastal Channel and Loire basins, predicted LDF showed significant changes as well. In the Loire basin, 15% of the sites presented a low predicted LDF (against 7% in initial conditions), with the major decreases of predicted LDF (up to 0.8) located in the medium part of the basin. However, 46% of the sites, mainly located in the upper part of the basin, remained unchanged with a high predicted LDF (> 0.8). Despite a few local changes, especially in the downstream part of the Rhine River, the North, the Seine, the Rhone, and the Garonne basins did not show significant changes in response patterns under the scenario 3. The scenario 4 induced local and punctual reductions in predicted LDF but did not significantly change the risks of taxon loss at the level of each large basin unit (Figs 6D and 7). At the whole France level, only the scenarios 2 and 3 induced a significant change in the predicted LDF (Fisher test p-value < 0.001).

## Discussion

### Decline in diadromous fishes

The first step of our work was to evaluate the loss of diadromous fishes in the French hydrographic network. Based on the analysis of a large range of historical sources we reconstructed the past distribution of species and we found that diadromous fishes occupied most of the main French rivers hundred years ago, illustrating the recent considerable loss of diadromous species at the national level. The Atlantic, English Channel and North Sea basins exhibited higher richness than the Mediterranean basin (e.g. the Rhone River). This has confirmed what is known about the historical-biogeographical distribution patterns of diadromous species [1, 55]. By comparing this past distribution with current data on taxon occurrences, we quantified the decline in diadromous taxon and found that this phenomenon is very widespread. Thus, according to our results, 45% of the 555 study sites, which were formerly inhabited by diadromous fishes, have currently lost their entire diadromous assemblage. Among the eight taxa studied, five have lost more than 50% of river length occupied two centuries ago in France. These observations are consistent with the massive decline in these species that has been observed elsewhere in Europe or eastern North America [7, 56, 57].

It should be noted that our estimate of diadromous loss is a conservative assessment that probably understates the real decline of some species occurrences. First, we considered occurrence data, but the persistence of a species may hide a potentially massive decline in terms of fish abundances. Second, we used relatively recent historical data (mainly from the late 18^th^ and 19^th^ centuries) to qualify the former species distribution, but it is well established that the human-induced decline in diadromous fishes started earlier and was already very significant in Western Europe since the Middle Ages [58, 59]. Third, despite an extensive effort in collecting historical data, the reconstruction of the past species distribution remains incomplete, either because existing historical sources have potentially been missed or simply because the past presence of some species was not recorded in old documents. In contrast, knowledge of the current distribution of species is much more exhaustive as it has involved many field experts and has been the subject of many inventories. As a result, the extinction or decline in diadromous assemblage was certainly underestimated in some areas of the river network. Fourth, the grouping of pairs of species under a single taxon (imposed by the limitations of historical data) could hide some extinction when both species initially coexisted but only one has remained. Finally, due to imprecisions regarding the location of some past occurrences, we have limited the presence of any given species to the lowest part of the watercourse, which has tended to underestimate the historical distribution of some species. To avoid misleading recommendations, stakeholders should keep all these limitations in mind if they would consider using our data and approach.

### Main drivers of loss

Our modeling approach has shown that the loss of diadromous fishes in the French river network can be largely explained by environmental conditions under anthropogenic pressures. Cross-validation suggests that the relationships highlighted between the variables and the loss of diadromous fishes are quite robust and of low sensitivity to possible outliers in the calibration dataset.

The present study confirmed that river fragmentation (i.e. from the sea to the considered site) is a major threat to diadromous fish [60–63]. In our model, statistics and the response curve associated with the maximum height of downstream barriers suggest that the size of the barriers is the factor that exerts the strongest and steepest effect on diadromous assemblage, leading to major and systematic impact when dams exceed about ten meters.

The density of barriers also contributes to diadromous assemblage degradation but seems to have a more diffuse and moderate effect than the size of downstream barriers. It reflects the potential cumulative effect of barriers. The cumulative effect of successive obstacles, such as small weirs or lock systems, that are not physically difficult to pass individually, may cause considerable problems to fish (e.g. delays, reduction in the migration success, injuries) [64–66]. According to its clearing rate, each barrier reduces the fraction of the community that can continue its migration. Even if the proportion of fish stuck in front of a given barrier is low, the succession of obstacles to cross can lead to a drastic reduction in the number of fish at the end of the course [26].

We also found a positive link between the distance from the sea and diadromous taxon loss suggesting that the most distant assemblages from the sea are intrinsically the most vulnerable. Indeed, with the distance, the energetic costs of migration rise as well as the risk of predation. However, it should be stressed that for a given density of barriers, a greater distance to the sea means more obstacles to be crossed for fish. In the context where practically all the French rivers are concerned by the presence of weirs or dams, the distance from the sea could also be considered as a descriptor of the cumulative detrimental impact of obstacles on diadromous fishes.

The close links observed between the loss of diadromous fishes and the height and density of barriers suggest that dam construction has had a significant contribution to the long-term decline in diadromous fishes. However, this result raises a certain paradox insofar as a large proportion of barriers referenced in the current ROE database, probably already existed when first historical observations of diadromous taxa were made. In Western Europe and particularly in France, the development of watercourses is several centuries old and expanded considerably in the second half of the Middle Ages with the widespread installation of watermills on most of the small and medium-sized rivers [58, 59, 67]. The number of mills has been estimated at 100,000 along French rivers in the 13^th^ century. This number had remained almost unchanged until 1809 when their use gradually ceased over the 19^th^ century [68]. The cessation of milling activities has not implied, however, the systematic disappearance of associated obstacles. Many of the current weirs and low head dams are legacies of medieval constructions (see for example Rouillard et al. [69] for the Seine River basin). Nevertheless, it would seem that the changes induced over time regarding (1) the management of these facilities (e.g. stopping the regular opening of gates previously required for the proper functioning and maintenance of the mills) and (2) their modernization (e.g. reinforcement, raising heights, design change) have made their passage more difficult for fish [70–73]. However, following technical progress, most of the medium to large dams were built later [20]; i.e. (1) during the 19^th^ century with the development of inland navigation and industrial expansion, and (2) after the Second World War as a consequence of economic development [20, 68]; for example, in France, 86% of the existing large dams (i.e., equal or higher than 15 m) were commissioned after 1930 [74]. Thus, most of the medium to large dams, which are the most impactful on diadromous fishes according to our results, was built after the period over which the historical distribution of diadromous fishes was described.

In accordance with the concept of extinction debt, the impact of barriers on fish assemblage can also extend over long periods of time, leading to a gradual decline in populations to their possible extirpation long after the obstacle construction [75]. For example, in a study examining the freshwater fish distribution in Japan, Fukushima et al. [61] have shown that the presence of dams negatively affected diadromous species and that, for some species, the duration since dam construction was also a relevant explicative variable. This suggests that the negative consequences of dam construction on fish populations involve long-term and gradual adverse processes. Thus, even if most of the barriers considered in our study had been already in place several centuries ago, i.e. before the period chosen to characterize the historical composition of diadromous fish assemblage, it is quite plausible that their effects persisted much later and could have contributed to the extinctions observed during the last two centuries. Integrating the construction date of dams and weirs is certainly a crucial issue for a better understanding of the dynamics and drivers of diadromous fish loss. Unfortunately, given the considerable number of barriers involved, this purpose was clearly beyond the scope of our present work.

Despite contrasting situations (from 0 to 100% of downstream barriers equipped), the ratio of barriers with fish passes was not retained in our model, thus highlighting the difficulty to quantify the mitigating effect of fish passes on diadromous fish loss. This result was unexpected given that fish pass establishment is a widely supported measure to restore river continuity and diadromous populations and that observation-based evidence supports the relationship between the upstream recovery of diadromous fish and the implementation of dam crossing facilities [76]. Several reasons may explain the absence of significant biological responses to this variable. First, it should be pointed out that our way of considering the state of the diadromous assemblage (loss/conservation of half of the taxa) may blur more subtle changes that could be induced by the establishment of fish passes (increase in population size, recovery of some species only). Second, most devices target specific species (particularly salmonids and shads) and stages (generally adults in upstream migration) and are very rarely effective for the whole diadromous assemblage and both upstream and downstream movements [77, 78]. Third, our description of fish passes did not take into account either their efficiency (some devices may be partially or totally ineffective due to their design or management) or their location in the hydrographic network (the impacts of fish passes are likely to be very different depending on whether they concern the most downstream dams or only dams in the upstream areas), two essential aspects concerning the effective recovery of populations [79]. Fourth, even if fish passes are locally effective, their succession along migration routes can generate cumulative effects that can jeopardize the maintenance of populations when significant numbers of obstacles need to be crossed [80, 81].

In addition to the variables related to river continuity, three variables that account for local alterations have been selected in our model suggesting that local degradations in water quality, hydrology, and river morphology also contributed to the loss of diadromous fishes. The hydrology alteration seems to have the strongest effect (based on the response curve and associated p-value). This result is in line with many studies demonstrating that changes in natural river flow may have detrimental effects on diadromous fishes especially through loss of stimuli for migration [82], loss of migration routes [78, 83, 84] and spawning grounds [85] or decreased survival of eggs and juveniles [64, 86]. Although the p-values associated with river morphology and water quality alteration were above the significance threshold, their inclusion in the model suggests that these variables improve the prediction of diadromous fish loss. Damages to river morphology may be particularly harmful when they affect spawning grounds and development areas for young stages [9, 16, 87–89]. Similarly, the abundant literature illustrating the decline of diadromous stocks due to pollution confirms this result [6, 14, 90, 91]. It should be noted that we addressed the water quality, river morphology and hydrology effects at a local scale only. But these alterations can be widespread in watersheds and generate river fragmentation undermining diadromous species, especially in case of water pollution [75]. This issue may be particularly detrimental to diadromous fishes when severe pollution usually affects the lower reaches of the river system, and forms a chemical barrier against migration due to cumulative effects along the continuum, largely preventing the movement of species further upstream (chemical barrier to migration [91, 92]).

The basin effect reveals that some basins are more prone to diadromous loss than others, regardless of barriers and local alterations already taken into account. This difference in vulnerability among basins can result from biogeographical differences in the distribution of diadromous fishes (for example, diadromous salmonids are naturally lacking in rivers flowing into the Mediterranean Sea). Given that all diadromous species are not equally sensitive to anthropogenic threats, basins naturally hosting the most sensitive species are expected to present diadromous assemblage with a higher risk of extinction. This difference in basin vulnerability could also result from natural conditions. For example, based on the intercepts, we found that the coastal Mediterranean basin was more vulnerable to diadromous losses. Yet, this basin essentially includes small watercourses exhibiting steep slopes in their upstream parts and a very harsh climatic context. Under such conditions, the suitable areas for diadromous species are of little extent, hosting small-sized populations, making them intrinsically more vulnerable to extinction. We can also assume that this basin effect accounts for other factors such as the impact of fisheries or long-term hydroclimatic changes, which were not directly integrated into our model.

Since river and marine catch data may only reflect the stock status and are not proof of overexploitation, testing the implication of fisheries in diadromous fish decline is a tricky task [93]. For this reason and due to a lack of data, we did not include this information into the model. However, even if we are not aware of any case in which overfishing has led directly to species extinction, overfishing may have contributed to the decline of some diadromous populations [94]. As an example, in France, European sturgeons were massively caught for caviar until the fishing ban in 1982, which certainly participated in the near extinction of this species [7, 95, 96]. Recruitment overfishing in the 20^th^ has also been pointed out as a reason for the dramatic drop in allis shad populations [97, 98] and salmons [6].

### Effect of different scenarios on richness loss

Based on four independent scenarios, we evaluated how the reduction in various anthropogenic pressures could reduce the proportion of the diadromous fish loss. These scenarios did not seek to evaluate realistic restoration plans but rather to clarify the main limitations to the recovery of diadromous assemblage. Moreover, the relevance of these scenarios for restoration purposes implies a certain resilience of diadromous fish populations to recover after the removal of some factors that led to their decline, an issue that is still under debate. Indeed, on the one hand, examples of dam removal or treatment of large-scale water pollutions, which were blocking for fish migration, have led to some noticeable successful recoveries of several species [99, 100], illustrating the strong natural recolonization capacity of most diadromous species [101]. On the other hand, some ambitious restoration plans implementing both intensive measures on habitats and extensive restocking have resulted in disappointing results or even failures [102].

The first lesson that can be drawn from the application of these scenarios is that measures focused exclusively on improving local conditions should have much more limited effects than those focused on restoring continuity. In contrast to the scenarios addressing river continuity, the scenario 4, based on the reduction in local alterations of water quality, river morphology, and hydrology, did not bring out widespread changes at both basin and country levels, and for sites showing signs of improvements, these were always of very limited amplitude. It should be noted, however, that even if we have considered river alterations and the presence of barriers independently, the removal of dams may result in local improvements of water quality, river morphology, and hydrology. The effect of improving local conditions can then be coupled with the effect of restoring continuity to consolidate the recovery of diadromous species. Given that local alterations may be reduced by barrier removals, the scenarios of barriers removals should have also integrated a reduction in local alterations. We did not integrate, however, this aspect in the modeling procedure, because we were not able to quantify a reduction in local alteration related to barrier removal.

Surprisingly, although our model revealed that the maximum height of dams has a considerable influence on diadromous fish loss, the scenario focusing on large dam removal (> 10 m, scenario 1) did not show the strongest responses. The relatively low consequences of this scenario are probably due to the small number of large dams (compared to lower ones) and their location, which, with a few exceptions, is restricted to the upper reaches. Thus, upstream sites concerned by large dams are generally also affected by many downstream smaller barriers that may themselves already have a negative impact on diadromous fish assemblage.

The two scenarios that predicted the most substantial and widespread responses addressed the removal of medium-sized (height between 2 and 10 m–scenario 2) or small-sized (height < 2 m–scenario 3) dams. The extent of the responses varied substantially between the two scenarios and among basins probably depending on the history of developments and the nature and location of existing dams and weirs specific to each basin. Thus, the Seine and North basins, whose main waterways have been largely developed with navigation facilities (e.g. weirs, locks) show greater improvements with the scenario 2, focusing on medium-size barriers. On the opposite, it is when removals focused on the small-size barriers (scenario 3) that responses are the most substantial for the Loire basin whose main axes are most often free-flowing, and for the coastal Atlantic and Channel basins. This suggests that the most effective measures for restoring the diadromous community are likely to vary significantly from one basin to another or from one region to another.

Overall, the outcomes from the various scenarios suggest that a large-scale improvement in diadromous fishes (e.g. assemblage throughout the French territory) will necessarily require large-scale measures to improve river continuity without limiting them to the largest dams but also taking into account smaller obstacles whose cumulative effect seems potentially very significant. The scenarios we have implemented are based on the removals of a considerable number of barriers but without considering their cost and effectiveness and their possible negative incidental consequences (e.g. loss of heritage and cultural value, economic and commercial impact, morphological destabilization). More refined approaches examining the cost/effectiveness balance could help to identify optimized scenarios, adapted to each basin and limiting the number of dams to remove while maintaining a good efficiency of the recovery of diadromous species [103].

### Implications for conservation and management

In this study, we used data on the historical and current fish distributions to quantify species loss of diadromous assemblage and help the identification of anthropogenic drivers that contributed to their long-term decline. Ignoring long-term ecosystem trends is likely to induce a shifting baseline syndrome that may lead to underestimating biodiversity losses and minimizing or ignoring the contribution of some of the anthropogenic drivers [104]. This concern is particularly acute in the case of diadromous fishes whose populations have long been severely depleted. By placing historical data, from one or two centuries ago, at the core of our approach, we have sought to limit this problem, but we cannot completely avoid it since the human-induced decline in diadromous fishes in Western Europe is a very old phenomenon dating back more than a millennium. This historical approach allowed us to highlight the negative impact of hindering the river continuity resulting from the construction of dams and weirs on diadromous assemblage. In this respect, it is noteworthy that some comparable statistical approaches, based solely on the current distribution of species, may have more difficulty to highlight the negative influence of barriers [55, 61]. However, using the past species occurrence to establish benchmarks for future restoration raises some issues, in the context of ongoing climate change. Prospective approaches predict significant changes in the continental distribution ranges of diadromous fishes due to warming and hydrological regime shifts [105]. Thus, basins now inhabited by certain species, particularly cold-water ones, could become unsuitable for them within a few decades. In this context and for effective and long-term restorations, historical occurrences of diadromous species should be seen as an indicator for potential recovery and not as a fixed list of species strictly defining future restoration targets.

## Supporting information

S1 File References for historical data. (DOCX)

S2 File Results of MCA computed on hydrological data. (DOCX)

S3 File Results of MCA computed on morphological data. (DOCX)

S4 File Results of MCA computed on water quality data. (DOCX)

S5 File Potential and current distribution maps per taxon. (DOCX)

S6 File Results of model selection (Table A) and model averaging approaches (Table B).
(DOCX)

S7 File Cross-validation metrics calculated on the calibration dataset (361 sites) and on the new datasets (30% (test dataset) and 70% (training dataset) of the randomly selected calibration sites). (DOCX)
