# Supplementary material for: Modeling diadromous fish loss from historical data: Identification of anthropogenic drivers and testing of mitigation scenarios
Source: PLoS One. 2020 Jul 28;15(7):e0236575. doi: 10.1371/journal.pone.0236575 (PMC7386633; doi:10.1371/journal.pone.0236575)
Supplement: S1 File — (DOCX) [file pone.0236575.s001.docx]

S1 File: References for historical data

ANJUBAULT, P. A. 1855. Revue des espèces de poissons qui vivent dans le département de la Sarthe. *Bulletin de la Société d'Agriculture, Sciences et Arts de la Sarthe,* XI**,** 258-287.

ANONYMOUS 1863a. [Capture of a sturgeon in the Loire River in 1811]. *Annales de la Société linnéenne du département de Maine-et-Loire,* 1863-1864**,** 78.

ANONYMOUS 1863b. Société d’horticulture et d’acclimatation du département de Tarn et Garonne. *Bulletin de la société impériale zoologique d’acclimatation* 10**,** 74.

ANONYMOUS 1889. Réponses au questionaire joint à la dépêche ministérielle du 27 décembre 1888, relative aux modifications à apporter à la législation sur la pêche au saumon. Ponts et Chaussées. Archives Nationales Paris F14 13603 Législation française sur la pêche du saumon (1889).

ANONYMOUS 1892. Fait divers et extraits de correspondance - la barbarie en économie piscicole sur le bord de la Garonne. *Bulletin de la Société Centrale d’Aquiculture et de Pêche,* 4**,** 205-206.

ANONYMOUS 1893. Pêche du saumon dans la Bidassoa. *Bulletin de la Société Centrale d’Aquiculture et de Pêche,* 5**,** 148.

ANONYMOUS 1894. Société de Pisciculture de la Creuse. *Bulletin de la Société Centrale d’Aquiculture et de Pêche,* 2**,** 157.

ANONYMOUS 1904. Esturgeon pris à Saint-Firmin-sur-Loire , le 26 mai 1904

ANONYMOUS 1920. Remonte du Flet. *Archives Nationales Fontainebleau. Versement 19920558 : Environnement, direction de la pêche. Art. 24*.

ANONYMOUS 1923. Remonte et fraye du Saumon en 1922-1923 -Cantonnement de Bayonne Ouest. Direction générale des Eaux et des Forêts. Archives Nationales.

ANONYMOUS 1924. Remonte et fraye du Saumon en 1923-1924- Pyrénées Atlantiques. Direction générale des Eaux et des Forêts. Archives Nationales.

ANONYMOUS 1930. Gros Poissons. *Bulletin Français de Pisciculture,* 20**,** 185-186.

ANONYMOUS 1932. Gros Poissons. *Bulletin Français de Pisciculture,* 43**,** 244.

ANONYMOUS 1933. Gros Poissons. *Bulletin Français de Pisciculture,* 56**,** 262.

ANONYMOUS 1934. Gros Poissons. *Bulletin Français de Pisciculture,* 68**,** 226.

ANONYMOUS 1935. Gros Poissons. *Bulletin Français de Pisciculture,* 80**,** 230.

ANONYMOUS 1936. Gros Poissons. *Bulletin Français de Pisciculture,* 94**,** 226.

ANONYMOUS 1937. Gros Poissons. *Bulletin Français de Pisciculture,* 104**,** 206.

ANONYMOUS 1938. Gros Poissons. *Bulletin Français de Pisciculture,* 110**,** 106.

ANONYMOUS 1939. Gros Poissons. *Bulletin Français de Pisciculture,* 117**,** 159.

ANONYMOUS 1943. Gros Poissons. *Bulletin Français de Pisciculture,* 131**,** 90.

ARRIGNON, J. & DE SAUVERZAC, R. 1969. *Carte piscicole de Seine Maritime*. Paris: Conseil Supérieur de la Pêche.

BACHELIER, R. 1963. L'Histoire du saumon en Loire. *Bulletin Français de Pisciculture,* 211**,** 49-70.

BACHELIER, R. 1964. L'Histoire du saumon en Loire. *Bulletin Français de Pisciculture,* 212**,** 86-103.

BELLOC, E. 1898. Noms scientifiques et vulgaires des principaux poissons d’eau douce. *Bulletin de la Société Centrale d’Aquiculture et de Pêche,* 10**,** 233-304; 313-439.

BELTREMIEUX, E. 1864. Faune du département de la Charente-Inférieure. *Académie de la Rochelle - Section des Sciences Naturelles -Annales,* 1862-63 N°6**,** 1-94.

BERT, P. 1864. *Le Catalogue des Vertébrés de l'Yonne* Paris V, Masson et Fils.

BLANCHARD, E. 1866. *Les poissons des eaux douces de la France: anatomie, physiologie, description des espèces, mŒurs, instincts, industrie, commerce, ressources alimentaires, pisciculture, législation concernant la pêche*, Baillière.

BLANCHARD, E. 1880. Les poissons des eaux douces de la France. Anatomie, physiologie, description des espèces, mœurs, instincts, industrie, commerce, ressources alimentaires, pisciculture. *J.B. Baillière (Ed)* 656pp.

BORODINE, N. 1892. L’établissement de pisciculture de Bergerac. *Bulletin de la Société Centrale d’Aquiculture et de Pêche,* 4**,** 209-213.

BOYER, H. 1930. *Le Saumon dans le Haut-Allier : sa vie, sa destruction, sa pêche.*, L'Ancre d'or.

BROCCHI, P. 1889. Note sur les causes qui ont amené la disparition du saumon dans la Meuse française. *Bulletin de la Société Centrale d’Aquiculture et de Pêche,* 1**,** 64-68.

BROCCHI, P. 1892. Le saumon ordinaire (Salmo salar) - Observations sur les moeurs. *Bulletin de la Société Centrale d’Aquiculture et de Pêche,* 4**,** 65-82; 101-123; 3 pl.

BROCCHI, P. 1894. Note sur la pisciculture dans le département de l'Isère. *Bulletin de la Société Centrale d’Aquiculture et de Pêche,* 6**,** 97-105.

BROU, P. 1953. Principales espèces de poissons existant dans le département de l'Allier. *Bulletin Français de Pisciculture,* 170**,** 38-40.

BUREAU, L. 1891. Le saumon de la Loire dans ses rapports avec la réglementation de la pêche. *Bulletin de la Société des Sciences naturelles de l'Ouest de la France,* 1891**,** 8-19.

CHIMITS, P. 1936. Remarques sur le saumon de l’Adour. *Bulletin Français de Pisciculture,* 99,100**,** 57-68, 92-98.

CHIMITS, P. 1950. *Carte piscicole du département des Landes*, 1/200000. Fédération des Associations de Pêche et de Pisciculture des Landes.

CHIMITS, P. 1954. *Carte piscicole de cours d'eau du bassin de l'Adour, Béarn et Pays basque*, 1/250000.

COUDERC, J. M. Etude ichtyogéographique de la Tourraine. 93 e Congrès national des sociétés savantes 5-9 avril 1968 1970 Tours. Actes du 93e Congrès national des sociétés savantes, Section de géographie, 137-174.

CRESPON, J. 1844. *Faune méridionale ou Description de tous les animaux vertébrés, vivants et fossiles, sauvages ou domestiques qui se rencontrent toute l'année ou qui ne sont que de passage dans la plus grande partie du Midi de la France; suivie d'une Méthode de taxidermie ou l'Art d'empailler les oiseaux. ,* Nîmes, Imprimerie Ballivet et Fabre.

CUILLER 1908. Sur les échelles à poissons du système Camére établies aux barrages verticaux des rivières l’Hyères et l’Aulne, faisant partie du canal de Nantes à Brest dans le Finistère. *Bulletin de la Société Centrale d’Aquiculture et de Pêche,* 20**,** 129-138.

CUVIER, G. & VALENCIENNES, A. 1847. *Histoire naturelle des poissons Tome 20*, P. Bertrand, Editeur.

CUVIER, G. & VALENCIENNES, A. 1848. *Histoire naturelle des poissons Tome 21*, P. Bertrand, Editeur.

DALPHONSE, F. J. B. 1803. *Mémoire statistique du département de l'Indre*, Imprimerie de la République.

DARLET, M. & PRIOUX, G. 1950. L’esturgeon et le caviar français. *Bulletin Français de Pisciculture,* 158**,** 5-13.

DAVOUST, F. 1855. Liste des poissons observés dans les eaux de la Vègres depuisBrûlon jusqu'à son embouchure. *Bulletin de la Société d'Agriculture, Sciences et Arts de la Sarthe,* XI**,** 234-236.

DE BOISSET, L. 1948. *Poissons des rivières de France: histoire naturelle pour les pêcheurs*, Librairie des Champs-Élysées.

DE BOMARE, J. C. V. 1791. *Dictionnaire Raisonné Universel D'Histoire Naturelle: Contenant L'Histoire Des Animaux, Des Végétaux Et Des Minéraux, et celle des Corps célestes, des Météores, et des autres principaux Phénomenes de la Nature: Avec L'Histoire Des Trois Regnes, et le détail des usages de leurs productions dans la Médecine, dans l'Économie domestique & champêtre, et dans les Arts & Métiers; Et une Table concordante des Noms Latins, etc. et le renvoi aux objets mentionnés dans cet Ouvrage. Tha-Zyz*, Bruyset.

DE KERVILLE, H. G. 1897. Faune de la Normandie. Fascicule 4. Reptiles, batraciens et poissons. *Bulletin de la Société des Amis des Sciences Naturelles de Rouen,* 4**,** 272-273.

DE LA TURTAUDIÈRE, P. A. M. 1828. *Faune de Maine et Loire, ou, Description méthodique des animaux qu'on rencontre dans toute l'étendue du département de Maine et Loire, tant sédentaires que de passage: avec des observations sur leurs mœurs, leurs habitudes, etc., etc.,* Angers, Paris, Rosier.

DE LACHADENÈDE, M. S. 1931. Le saumon dans les Gaves et les échelles à poissons. *Bulletin Français de Pisciculture,* 40**,** 97-102.

DE LACHADENÈDE, M. S. 1935. Echelles à saumons et résultats obtenus sur les Gaves. *Bulletin Français de Pisciculture,* 85**,** 5-10.

DE LACHADENÈDE, M. S. 1958. Les Gaves, les saumons, les échelles. *Bulletin Français de Pisciculture* 190**,** 13-24.

DE SELYS-LONGCHAMPS, E. 1842. *Faune belge, 1re partie, Indication méthodique des mammifères, oiseaux, reptiles et poissons observés jusqu'ici en Belgique: i-xii, 1-310–Dessain, Liège/C,* Muquart, Bruxelles, Dessain, M.

DE SINÉTY, E. 1855. Notes pour servir à la Faune du département de Seine-et-Marne, ou liste méthodique des animaux vivants à l'état sauvage qui se rencontrent, soit constament, soit périodiquement ou accidentellement, dans ce département. *Extrait de la Revue et Magasin de Zoologie* 2e série Tome VII**,** 231-238.

DE SOLAND, A. 1869. Etude sur les poissons de l'Anjou. Angers, France: Imprimerie P. Lachese, Belleuvre et Dolbeau.

DE VILLENEUVE, C. 1821. *Statistique du département des Bouches-du-Rhône, avec Atlas,* Marseille, Antoine Ricard.

DEL PERE DE CARDAILLAC DE SAINT-PAUL, G. 1902. Quelques observations sur la question des barrages et des échelles à poissons. *Bulletin de la Société Centrale d’Aquiculture et de Pêche,* 14**,** 265-271.

DELARBRE, A. 1798. *Essai zoologique sur l'Auvergne, ou histoire naturelle des animaux quadrupèdes, et oiseaux indigènes ; de ceux qui ne sont que passagers ou qui parraissent rarement, et des poissons et amphibies, observés dans cette province.,* Paris, Gugour, A. J.

DESVAUX, A. N. 1851. *Essai d'ichtyologie des côtes océaniques et de l'intérieur de la France,* Anger, Cosmé et Lachèse.

DORIER, A. 1954. *Carte piscicole du département de la Drôme*. Grenoble: Imp. Allier.

DOTTRENS, E. 1951. *Poissons d'eau douce: I. Des Lamproies aux Salmonidés,* Neuchâtel et Paris, Delachaux & Niestlé.

DROUIN DE BOUVILLE, R. 1930. Observations sur le saut du saumon. *Bulletin Français de Pisciculture,* 25**,** 5-8.

DROUIN DE BOUVILLE, R. 1941. Le saumon revient-il à sa frayère natale ou, tout au moins, à son bassin fluvial d'origine? *Bulletin Français de Pisciculture,* 123**,** 10-27.

DU MONCEAU, H. L. D. 1769. *Traité Général des Pesches, et Histoire des Poissons: qu'elles fournissent, tant pour la subsistance des hommes, que pour plusieurs autres usages qui ont rapport aux Arts et au Commerce*, Saillant & Nyon.

DU MONCEAU, H. L. D. 1776. *Traité Général des Pesches, et Histoire des Poissons: qu'elles fournissent, tant pour la subsistance des hommes, que pour plusieurs autres usages qui ont rapport aux Arts et au Commerce*, Imprimerie de L. F. Delatour.

DUBOIS, P. 1903. Les poissons du département du Cher d’après la classification du Dr. Moreau. *Bulletin de la Société Centrale d’Aquiculture et de Pêche,* 15**,** 53-59.

DUMÉRIL, A. H. A. 1865. *Histoire naturelle des poissons ou ichthyologie générale. Atlas.,* Paris, Librairie Encyclopédique de Roret.

DURAND, R. 1963a. *Carte de l'hydrographie et de l'hydrobiologie piscicole du département de Meurthe et Moselle*. Anger: Imp. H. Siraudeau.

DURAND, R. 1963b. Contribution à l'étude de l'hydrographie et de l'hydrobiologie piscicole du département de Meurthe et Moselle. Anger.

EUZENAT, G., PÉNIL, C. & ALLARDI, J. 1992. Migr'en Seine. Stratégie pour le retour du saumon en Seine. SIAAP - Conseil Supérieur de la Pêche.

FÉDÉRATION DES ASSOCIATIONS DE PÊCHE ET PISCICULTURE DU FINISTÈRE 1948. L'Aulne et la pêche au saumon. *Bulletin Français de Pisciculture,* 148,149**,** (supplément).

FOURNEL, D. H. L. 1836. *Faune de la Moselle,* Metz, Verronnais.

GALLOIS, C. 1945. Eclusage d'Aloses. *Bulletin Français de Pisciculture,* 137**,** 209-210.

GALLOIS, C. 1946-1947. L’alose du Rhône. *Bulletin Français de Pisciculture,* 141-144**,** 162-176, 5-14, 72-79, 130-136.

GALLOIS, C. 1947a. Aménagement du Rhône vivarais par la Compagnie Nationale du Rhône; ses incidences sur l'économie piscicole, mesures tendant à les atténuer. *Bulletin Français de Pisciculture,* 146**,** 25-34.

GALLOIS, C. 1947b. La fécondation artificielle de l’alose du Rhône. *Bulletin Français de Pisciculture,* 145**,** 198.

GAYOU, F. 1984. Note sur "historique des poissons migrateurs de la Garonne. *Rapport CSP***,** 3 P.

GÉHIN, J. B. 1868. Révision de poissons qui vivent dans les cours d'eau et dans les étangs du département de la Moselle avec quelques considérations sur le darwinnisme. *Bulletin de la Société d'Histoire Naturelle du département de la Moselle,* Onzième cahier**,** 139-242.

GENSOUL, J. 1908. Monographie des poissons du département de Saône-et-Loire. *Bulletin de la Société d'Histoire Naturelle d'Autun***,** 153-247.

GENTIL, A. 1883. Ichtyologie de la Sarthe. *Bulletin de la Société d'Agriculture, Sciences et Arts de la Sarthe,* XXIX**,** 356-379.

GERVAIS, H. F. P. & BOULART, R. 1897. *Les poissons d'eau douce: synonymie, description, mœurs, frai, pêche, iconographie, des espèces composant plus particulièrement la faune française. ,* Paris, J. Rothschild.

GISCARD, G. 1930. Le problème du Saumon en Garonne. *Toulouse, Imp. Sud-. Ouest,* **,** 48 p.

GODRON, D. A. 1862. *Zoologie de la Lorraine. Ou catalogue des animaux sauvages observés jusqu'ici dans cette ancienne province,* Nancy, Raybois.

GREGOIRE, P. 1983. *Contribution a la connaissance de la répartition des poissons d’eau douce en France. .* DEA d’ecologie de l’universite Paris VI. Paris.

HOESTLANDT, H. 1971. *Carte Piscicole du département du Pas-de-Calais*.

KEITH, P. 1995. Cinq exemples d'évolutions de populations piscicoles. Paris: RNDE, Secrétariat de la faune et de la flore-Muséum National d'Histoire Naturelle, Conseil Supérieur de la Pêche, Ministère de l'Environnement.

KEITH, P. 1998. *Evolution des peuplements ichtyologiques de France et stratégies de conservation.* Rennes 1.

KIENER, A. 1985. *Au fil de l'eau en pays méditerranéen: milieux aquatiques, poissons et pêche, gestion.,* Avignon, Aubanel.

LAHILLE, F. 1888. Les poissons de Toulouse et des environs. *Bulletin de la Société d'Histoire Naturelle de Toulouse,* 1888**,** LIX - LXIII.

LAMBERT 1894. Une nouvelle station de la grémille *Acerina cernua* Cuvier. Son apparition dans la rivière de la Mayenne, suivie d'une liste des poissons signalés jusqu'à ce jour dans le département de la Mayenne.

. *Bulletin de la Société des Sciences Naturelles de l'Ouest de la France,* 1894**,** 10-16.

LARRIEU, J. 1939. L’équipement piscicole du bassin de l’Adour. *Bulletin Français de Pisciculture,* 118**,** 174-179.

LARRIEU, J. 1941. De l’efficacité des échelles ou passes à saumons. *Bulletin Français de Pisciculture,* 123**,** 5-9.

LATASTE, F. 1894a. *Actes de la Soc. scient. du Chili, vol IV***,** LX.

LATASTE, F. 1894b. Allures bizarres d’un esturgeon dans la Garonne. *Bulletin de la Société Centrale d’Aquiculture et de Pêche,* 6**,** 271.

LAVOLLÉE, G. 1902. Le saumon en Seine. *Bulletin de la Société Centrale d’Aquiculture et de Pêche***,** 221-234.

LAVOLLÉE, G. 1903. Bassin de la Seine. Enquête sur les mesures à prendre pour assurer le passe des poissons migrateurs. *In:* NATIONALES, A. (ed.). Paris F14 13618.: Rapport de l'Ingénieur en Chef chargé de suivre les enquêtes dans le bassin de la Seine

LE CLERC, J. 1941. Note sur des essais de multiplication artificielle de l’alose dans le bassin de la Loire. *Bulletin Français de Pisciculture,* 123**,** 27-37.

LEBEL, I., MÉNELLA, J. Y. & LE CORRE, M. 2001. Bilan des actions du plan migrateurs concernant l’alose feinte (Alosa fallax rhodanensis) sur le bassin Rhône-Méditerranée-Corse. *Bulletin Français de la Pêche et de la Pisciculture,* 362-363**,** 1077-1100.

LEGAGNEUR, J. & ARRIGNON, J. 1976. *Carte piscicole du département de la Somme*. Conseil Supérieur de la Pêche.

LÉGER, L. 1926. *Carte piscicole du département de l'Ain*. Grenoble: Lith Alliaer.

LÉGER L. & BURDIN, A. 1945. *Carte piscicole du département du Rhône*. Grenoble.

LEMARIE, E. 1866. Poissons des départements de la Charente, de la Charente Inférieure, des Deux-Sèvres, de la Vendée et de la Vienne. *Mémoires de la société de Statistique, Siences et Arts des deux-Sèvres,* 2^me^ Série Tome VI**,** 74-114.

LETACQ, A. L. 1896. Matériaux pour servir à la faune des vertébrés du département de l'Orne. *Annuaire des cinq départements de la Normandie,* 62**,** 67-130.

LETACQ, A. L. 1906. Sur les Mammifères, les Oiseaux et les Poissons disparus ou en voie de disparition de la faune de l'Orne. *Bulletin de la Societe Linneenne de Normandie,* 9**,** 52-73.

MAGNIN, E. 1959. Détermination de l'âge et croissance de l'*Acipenser sturio* L. de la Gironde. . *Bulletin Français de Pisciculture* 193**,** 152-159.

MAGNIN, E. 1963. Recherches sur la systématique et la biologie des Acipenséridés Acipenser sturio L'Acipenser oxyrhynchus Mitchill, Acipenser fulvescens Raf *Annales de la Station Centrale d'Hydrobiologie Appliquée (Paris)***,** 8-242.

MARCOTTE, F. 1860. Les animaux vertébrés de l'arrondissement d'Abbeville. *Mémoires de la Société Impériale d'Emulation d'Abbeville,* 2e série 9e volume**,** 217-470.

MARTIN, R. 1894. Le saumon commun dans le département de l’Indre. *Bulletin de la Société Centrale d’Aquiculture et de Pêche,* 6**,** 268-270.

MARTIN, R. & ROLLINAT, R. 1892. Catalogue des reptiles, batraciens et poissons du département de l'Indre. *Mémoires de la Société Zoologique de France,* Tome V**,** 30-45.

MARTIN, R. & ROLLINAT, R. 1914. *Description et mœurs des mammifères, oiseaux, reptiles, batraciens et poissons de la France centrale.,* Paris, P.Lechevalier.

MATHIAS, P. 1929. Sur un trématode parasite des aloses (Pronopyge ventricosum, (Rudolphi)). *Bulletin de la Société Centrale d’Aquiculture et de Pêche,* 36**,** 103-110.

MAUDUYT, F. 1849. Ichthyologie de la Vienne, ou tableau méthodique et descriptif des poissons qui vivent habituellement dans les eaux de ce département ou qui y remontent périodiquement et accidentellement. *Bulletin de la Société Académique d'Agriculture belles-Lettres, Sciences et Arts de Poitiers***,** 8-49.

MAUDUYT, F. 1851. Ichthyologie de la Vienne. *Bulletin de la Société Académique d'Agriculture belles-Lettres, Sciences et Arts de Poitiers***,** 163-176.

MELLEVILLE, M. 1863. *Dictionnaire Historique du département de l'Aisne*.

MERGOT, FERBOEUF & MARCHAIS. 1950. *Carte piscicole du département d'Indre-et-Loire au 200.000^e^*. Tours: Fédération de Pêche d'Indre et Loire.

MINISTÈRE DE L'AGRICULTURE & DIRECTION GÉNÉRALE DES EAUX ET FORETS 1930. Etudes sur les mesures à prendre pour assurer la circulation et la conservation des poissons migrateurs dans les cours d'eau faisant l'objet de concessions d'énergie hydraulique. *in : Annales, fasc.58.* Paris: Imprimerie Nationale.

MOREAU, E. 1881. *Histoire naturelle des poissons de la France,* Paris, G. Masson.

MOREAU, E. 1897. Les poissons du département de l'Yonne. *Bulletin la Société des Sciences Historiques et Naturelles de l'Yonne,* 51**,** 143-227.

MOREAU, E. 1898. Les poissons du département de l'Yonne (suite). *Bulletin la Société des Sciences Historiques et Naturelles de l'Yonne,* 51**,** 3-82.

MOUSSIER 1853. Catalogue des animaux vertébrés, observés dans le département de la Haute-Loire, et composant une grande partie des collections zoologiques du musée du Puy. *Annales de la Société d'agriculture, sciences, arts et commerce du Puy,* 1853 Tome XVIII - 31**,** 373-450.

NOULET, J. B. 1891. Liste des poissons de la Haute-Garonne et synonymie locale. *Revue des Pyrénées et de la France méridionale,* Tome III**,** 429-433.

OGÉRIEN, F. & MICHALET, E. 1863. *Histoire naturelle du Jura et des départements voisins,* Paris, Lons-Le-Saunier, Victor Masson et Fils.

PARÂTRE, R. 1894a. Du dépeuplement des cours d’eau de l’Indre. *Bulletin de la Société Centrale d’Aquiculture et de Pêche,* 6**,** 1-30.

PARÂTRE, R. 1894b. Faune du Doubs (mammifères, reptiles, batraciens et poissons) par E. Olivier. *Bulletin de la Société Centrale d’Aquiculture et de Pêche,* 6**,** 7-9.

PARÂTRE, R. 1894c. Vertébrés sauvages du département de l’Indre par R. Martin et R. Rollinat. *Bulletin de la Société Centrale d’Aquiculture et de Pêche,* 6**,** 239-251.

PAULZE D'IVOY DE LA POYPE 1901. La question de la Pêche dans le Bassin de la Loire. Poitiers.

PAULZE D'IVOY DE LA POYPE 1906. La pêche en France- le bassin de l’Adour. *Bulletin de la Société Centrale d’Aquiculture et de Pêche,* 18**,** 212-219.

PAULZE D'IVOY DE LA POYPE 1912. Etude scientifique des écailles de six saumons français. *Bulletin de la Société Centrale d’Aquiculture et de Pêche,* 24**,** 17-20.

PAULZE D'IVOY DE LA POYPE 1914. La croissance du saumon français d’après l’étude de ses écailles. *Bulletin de la Société Centrale d’Aquiculture et de Pêche,* 26**,** 61-62.

PEDROLI, J. C., ZAUGG, B. & KIRCHHOFER, A. 1991. *Atlas de distribution des poissons et cyclostomes de Suisse,* Neuchâtel, Schweizerisches Zentrum für die kartografische Erfassung der Fauna.

PICHON & TOURNIOL 1864. Lettre adressée par MM. Pichon et Tourniol, propriétaires à Milianah, à M. le Président de la société impériale d’acclimatation. *Bulletin mensuel de la Société Imperiale Zoologique d'Acclimatation,* 1**,** 435-438.

PITON, L. 1931. Faune d'Auvergne. Poissons (suite). *Bulletin de la Société d'Histoire Naturelle d'Auvergne,* 18-19**,** 27-47.

PITON, L. 1932. Faune de l'Auvergne. Poissons (fin). *Bulletin de la Société d'Histoire Naturelle d'Auvergne,* 20**,** 43-71.

POPLIN, R. 1952. Le peuplement des eaux de l’Yonne moyenne. *Bulletin Français de Pisciculture,* 164**,** 109-114.

RAY, J. 1851. *Rapport sur la pêche fluviale dans le département de l'Aube,* Troyes, Soc. agric. Sci. Arts et Belles Lettres de l'Aube.

RAY, J., LEBASTEUR, CLÉMENT-MULLET, J. J., ANNER-ANDRÉ & GAYOT 1852. Rapport sur la pêche fluviale dans le département de l'Aube. *Mémoires de la société d'Agricultue Sciences et Arts de l'Aube,* 2^ème^ Série**,** 71-106.

RÉGUIS, J. M. F. 1881. Essai sur l'Histoire Naturelle des vertébrés de la Provence et des départements Circonvoisins. *Bulletin de la Société d'études scientifiques et archéologiques de la ville de Draguignan,* 1880-1881 Tome XIII**,** 3-430.

RÉGUIS, J. M. F. 1896. Faune ichtyologique de Vaucluse. *Mémoires de l'Académie de Vaucluse,* 1896**,** 196-233.

ROULE, L. 1913. Contribution à l’étude de la biologie du saumon. *Bulletin de la Société Centrale d’Aquiculture et de Pêche,* 25**,** 166-168.

ROULE, L. 1914. Sur l’influence exercée sur la migration de montée du saumon (Salmo salar L.) par la proportion d’oxygène dissous dans l’eau des fleuves. *C. R. Hebd. Seances Acad. Sci.,* 158**,** 1364-1366.

ROULE, L. 1920. *Etude sur le saumon des eaux douces de la France: considéré au point de vue de son état naturel et du repeuplement de nos rivières*, Imprimerie nationale.

ROULE, L. 1921. L’écologie actuelle du saumon Atlantique (Salmo salar L.) dans les cours d’eau de notre pays. *Bulletin de la Société Centrale d’Aquiculture et de Pêche,* 28**,** 55-58.

ROULE, L. 1923a. Notes sur les aloses de la Loire et de l’Aquitaine. *Bulletin de la Société Centrale d’Aquiculture et de Pêche,* 30**,** 14-22.

ROULE, L. 1923b. *Nouvelle contribution à l'étude de l'esturgeon (Acipenser sturio L.) dans l'Europe Occidentale et de sa diminution progressive,* Paris, Editions Blondel de la Rougery.

ROULE, L. & DROUIN DE BOUVILLE, R. 1927. Considérations sur la pénétration du Saumon (Salmo salar L.) dans les estuaires du Littoral de la France pour le début de sa migration de montée. *Bulletin de l'Institut Océanographique,* 504.

ROY, R. 1952. Biogéographie des poissons dans la région de Nevers. *in: Annales de la Station Centrale d’Hydrobiologie Appliquée* 4**,** 287-317.

SCHNEIDER, M. A. 1901. Le saumon mange-t-il en eau douce ? *Bulletin de la Société Centrale d’Aquiculture et de Pêche,* XIII**,** 264.

SOUBEIRAN, J. L. 1871. Rapport sur les expositions internationales de pêche de Boulogne-sur-Mer, Arcachon et du Havre (1866-1868). *Bulletin de la Société d’Acclimatation .* 8**,** p. 81-117; 168-218; 304-347; 401-458.

SPILLMANN, C. J. 1961. Faune de France 65 poissons d'eau douce. Paris: CNRS.

THIBAULT, M. & BILLARD, R. 1987. *La restauration des rivières à saumons,* Paris, Ed. INRA.

VALENCIENNES, A. 1863. Note sur les espèces de poissons qui peuvent être entretenus vivants dans les aquariums. *Bulletin de la Société Impériale Zoologique d’Acclimatation,* 10**,** 177-183.

VALLOT, J. N. 1836. Histoire naturelle des poissons du département de la Côte-d'Or. *Académie des Sciences Arts et Belles Lettres de Dijon***,** 5-310.

VALLOT, J. N. 1837. *Ichthyologie française, ou, Histoire naturelle des poissons d'eau douce de la France.,* Dijon, Imprimerie de E. Frantin.

VALLOT, J. N. 1850. *Supplément à l'Ichtyologie française, et Tableau général de poissons d'eau douce de la France,* Dijon, Tricault, E.

VIBERT, R. 1943. Protection du saumon - Possibilités de réacclimatation. *Bulletin Français de Pisciculture,* 128**,** 89-107.

VIBERT, R. 1945. Les poissons migrateurs dans l’économie piscicole du Sud-Ouest. *Bulletin Français de Pisciculture,* 136**,** 121-135.

VIBERT, R. 1948. Les poissons migrateurs dans l’économie piscicole française (Compte rendu de la commission technique des poissons migrateurs réunie le 7 juin 1948). *Bulletin Français de Pisciculture,* 151**,** 66-88.

VIBERT, R. 1950. Recherches sur le saumon de l'Adour (*Salmo salar*, Linné). Ages, croissance, cycle génétique, races, 1942-1948. *Annales de la Station Centrale d'Hydrobiologie Appliquée,* 3**,** 27-149.

VINCENT, P. 1890. Extrait d’un rapport adressé au ministère de l’agriculture sur l’organisation d’un établissement destiné à la reproduction artificielle de l’alose (reproduction de Vincent, P. 1889 Bull. Minist. Agric. décembre 1889). *Bulletin de la Société Centrale d’Aquiculture et de Pêche,* 2**,** 17-27.

VIOLETTE, A. 1902. La question du saumon. *Bulletin de la Société Centrale d’Aquiculture et de Pêche,* 14**,** 253-261.

VIVIER, P. 1940. Le peuplement piscicole des lacs subalpins de Savoie. *Bulletin Français de Pisciculture,* 120**,** 61-66.

VIVIER, P. 1959. Comité scientifique de la station centrale d’hydrobiologie appliquée (Le Paraclet, 26 mai 1959). *Bulletin Français de Pisciculture,* 32**,** 65.

WURTZ, A. 1963. *Carte Piscicole du département de la Somme*.

ZSCHOKKE, F. 1884. *Recherches sur l'organisation et la distribution zoologique des vers parasites des poissons d'eau douce. .* Thèse de doctorat : Univ. Genève.
