# Supplementary material for: Modeling diadromous fish loss from historical data: Identification of anthropogenic drivers and testing of mitigation scenarios
Source: PLoS One. 2020 Jul 28;15(7):e0236575. doi: 10.1371/journal.pone.0236575 (PMC7386633; doi:10.1371/journal.pone.0236575)
Supplement: S2 File — (DOCX) [file pone.0236575.s002.docx]

**S2 File: Results of MCA computed on hydrological data**

| 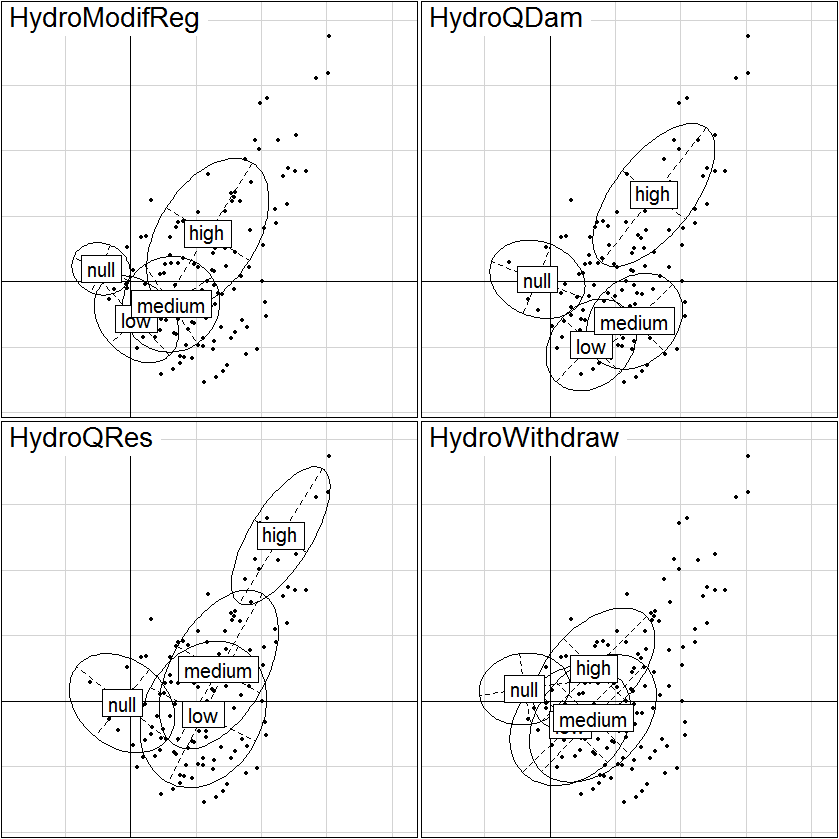 | \|  \| Axis1 \| Axis2 \| \| --- \| --- \| --- \| \| Eigenvalue \| 0.47 \| 0.34 \| \| Percentage of variance \| 15.76 \| 11.32 \| \| HydroModifReg \| 0.65 \| 0.48 \| \| HydroQDam \| 0.49 \| 0.46 \| \| HydroQRes \| 0.37 \| 0.16 \| \| HydroWithdraw \| 0.37 \| 0.25 \| |
| --- | --- | --- | --- | --- | --- | --- | --- | --- | --- | --- | --- | --- | --- | --- | --- | --- | --- | --- | --- | --- | --- | --- |

Legend :

| HydroModifReg: | Modified hydrological regime | |
| --- | --- | --- |
| HydroQdam: | Hydropeaking |  |
| HydroQres: | By-pass channel |  |
| HydroWithdraw: | Water abstraction |  |
