# Supplementary material for: Modeling diadromous fish loss from historical data: Identification of anthropogenic drivers and testing of mitigation scenarios
Source: PLoS One. 2020 Jul 28;15(7):e0236575. doi: 10.1371/journal.pone.0236575 (PMC7386633; doi:10.1371/journal.pone.0236575)
Supplement: S3 File — (DOCX) [file pone.0236575.s003.docx]

**S3 File: Results of MCA computed on morphological data**

| 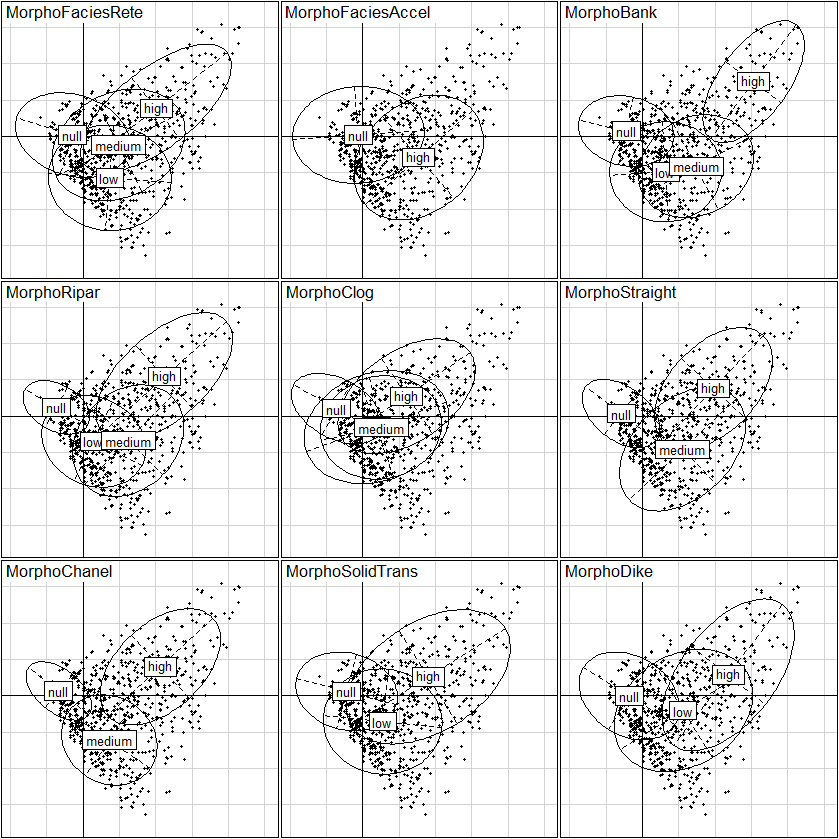 | \|  \| Axis1 \| Axis2 \| \| --- \| --- \| --- \| \| Eigenvalue \| 0.41 \| 0.21 \| \| Percentage of variance \| 17.6 \| 9.17 \| \| MorphoFaciesRete \| 0.28 \| 0.18 \| \| MorphoFaciesAccel \| 0.11 \| 0.03 \| \| MorphoBank \| 0.49 \| 0.36 \| \| MorphoRipar \| 0.58 \| 0.36 \| \| MorphoClog \| 0.27 \| 0.11 \| \| MorphoStraight \| 0.56 \| 0.25 \| \| MorphoChanel \| 0.66 \| 0.43 \| \| MorphoSolidTrans \| 0.34 \| 0.17 \| \| MorphoDike \| 0.41 \| 0.05 \| |
| --- | --- | --- | --- | --- | --- | --- | --- | --- | --- | --- | --- | --- | --- | --- | --- | --- | --- | --- | --- | --- | --- | --- | --- | --- | --- | --- | --- | --- | --- | --- | --- | --- | --- | --- | --- | --- | --- |

Legend :

| MorphoFaciesSlow: | Presence of an impoundment |
| --- | --- |
| MorphoFaciesAccel: | Instream habitat modified |
| MorphoBank: | Artificial embankment |
| MorphoRipar: | Riparian vegetation modified |
| MorphoClog: | Sedimentation |
| MorphoStraight: | Channel form modified |
| MorphoChanel: | Cross-section modified |
| MorphoSolidTrans: | Channel incision or aggradation |
| MorphoDike: | Diked |
