# Supplementary material for: Modeling diadromous fish loss from historical data: Identification of anthropogenic drivers and testing of mitigation scenarios
Source: PLoS One. 2020 Jul 28;15(7):e0236575. doi: 10.1371/journal.pone.0236575 (PMC7386633; doi:10.1371/journal.pone.0236575)
Supplement: S4 File — (DOCX) [file pone.0236575.s004.docx]

**S4 File: Results of MCA computed on water quality data**

| 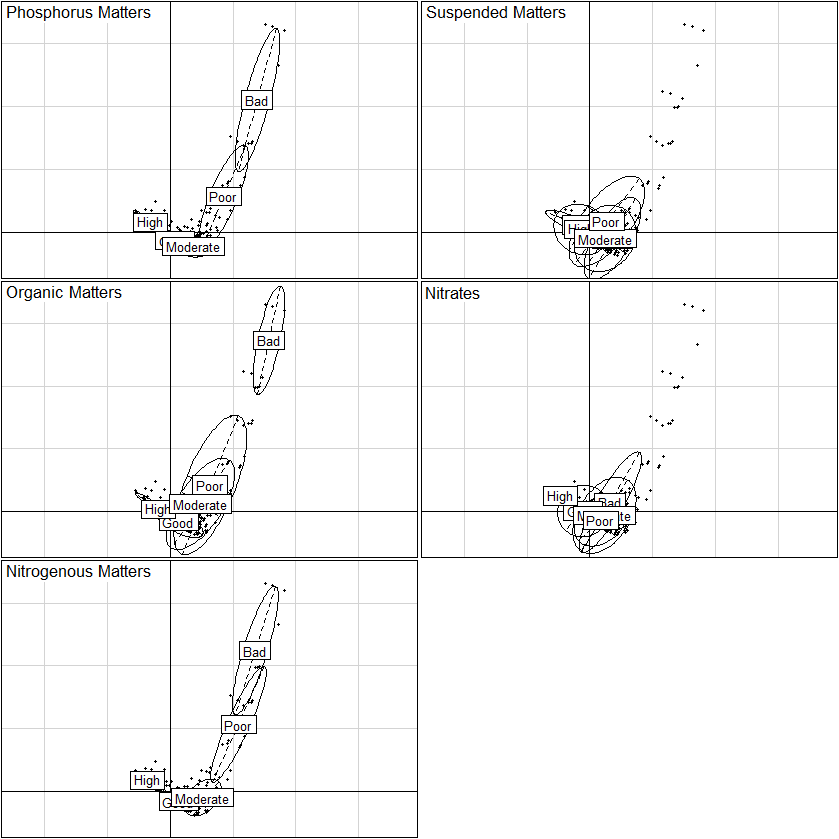 | \|  \| Axis1 \| Axis2 \| \| --- \| --- \| --- \| \| Eigenvalue \| 0.54 \| 0.43 \| \| Percentage of variance \| 13.49 \| 10.79 \| \| Phosphorus Matters \| 0.71 \| 0.74 \| \| Suspended Matters \| 0.34 \| 0.04 \| \| Organic Matters \| 0.48 \| 0.46 \| \| Nitrates \| 0.41 \| 0.11 \| \| Nitrogenous Matters \| 0.76 \| 0.81 \| |
| --- | --- | --- | --- | --- | --- | --- | --- | --- | --- | --- | --- | --- | --- | --- | --- | --- | --- | --- | --- | --- | --- | --- | --- | --- | --- |
|  |  |
