# Supplementary material for: Modeling diadromous fish loss from historical data: Identification of anthropogenic drivers and testing of mitigation scenarios
Source: PLoS One. 2020 Jul 28;15(7):e0236575. doi: 10.1371/journal.pone.0236575 (PMC7386633; doi:10.1371/journal.pone.0236575)
Supplement: S5 File — (DOCX) [file pone.0236575.s005.docx]

S5 File: Potential and current distribution maps per taxon.

ALT: shads - including allis shad (*Alosa alosa*) and twait shad (*Alosa fallax*); LPT: lampreys - including sea lamprey (*Petromyzon marinus*) and river lamprey (*Lampetra fluviatilis*); MUX: mullets - including thinlip grey mullet (*Liza ramada*) and thicklip grey mullet (*Chelon labrosus*); SAL: salmonids - including sea trout (*Salmo trutta trutta*) and Atlantic salmon (*Salmo salar*); STU: sturgeons including Atlantic sturgeon (*Acipenser oxyrinchus*) and European sturgeon (*Acipenser sturio*); FLE: European flounder (*Platichthys flesus*); COR: houting (*Coregonus oxyrinchus*); EPE: smelt (*Osmerus eperlanus*).

| 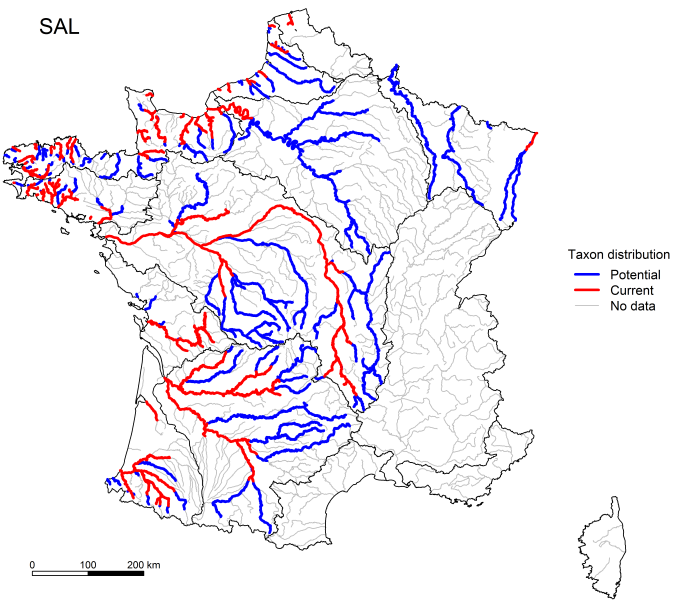 | 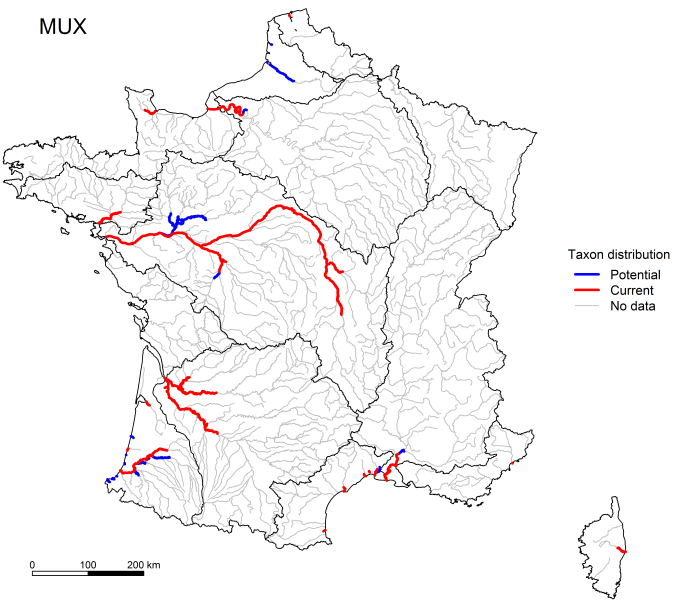 |
| --- | --- |
| 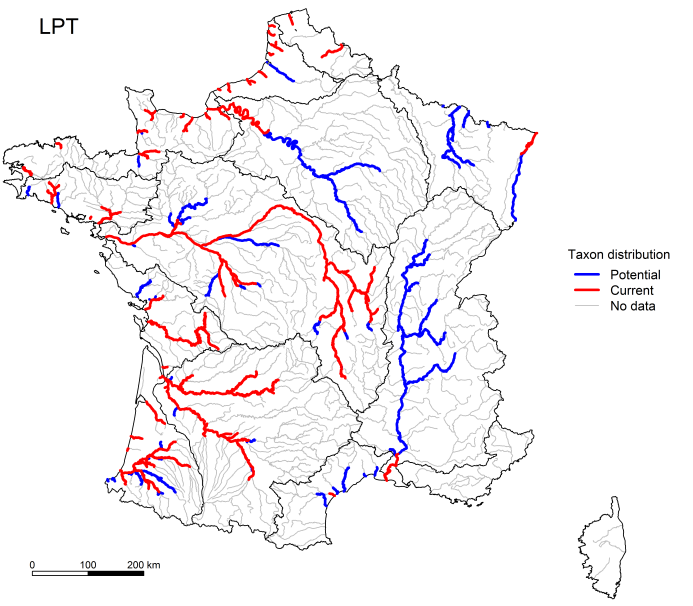 | 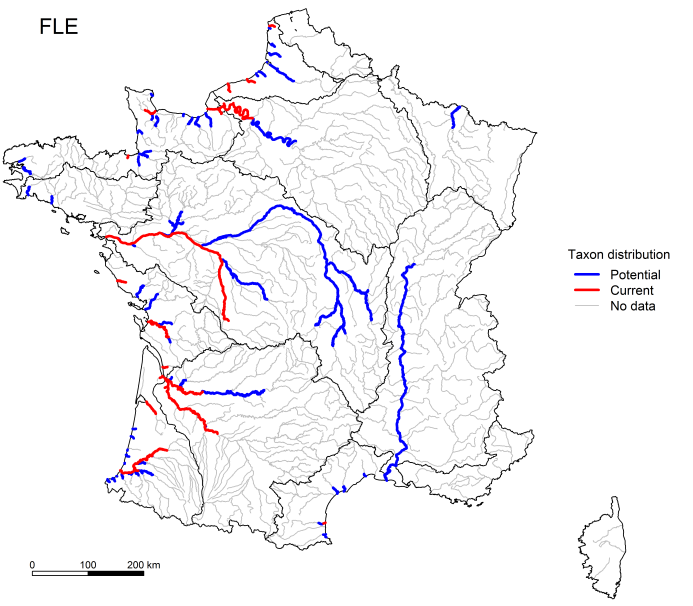 |
| 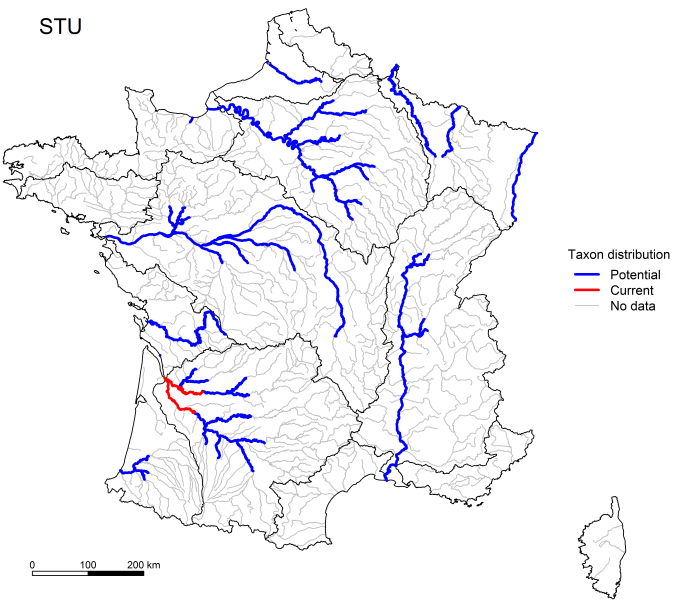 | 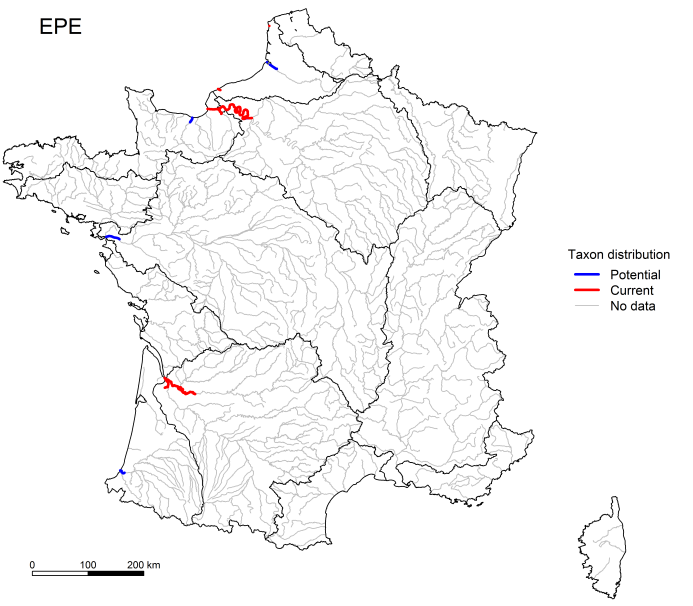 |
| 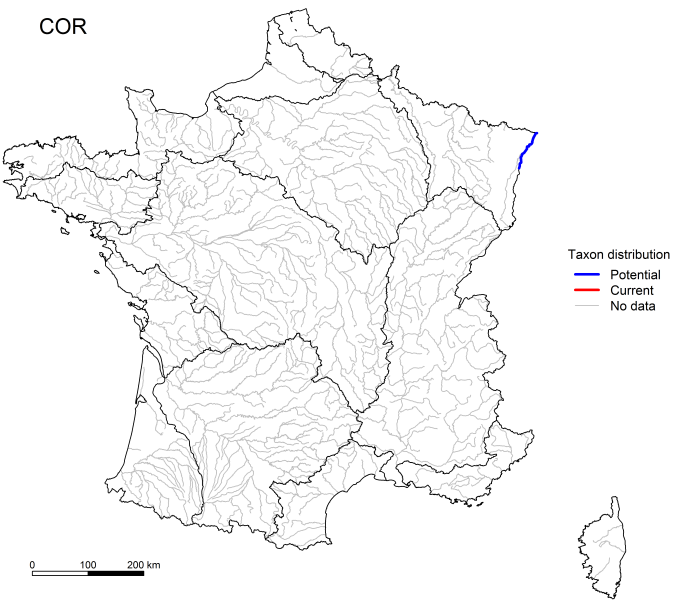 | 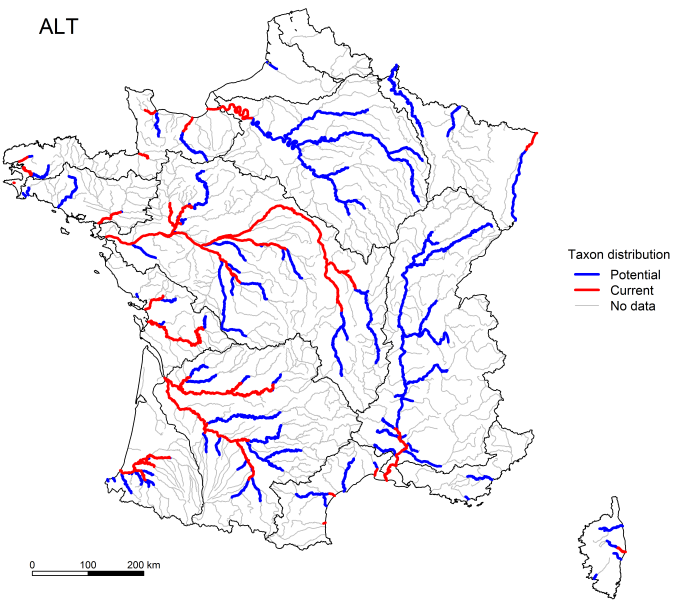 |
