# Supplementary material for: Modeling diadromous fish loss from historical data: Identification of anthropogenic drivers and testing of mitigation scenarios
Source: PLoS One. 2020 Jul 28;15(7):e0236575. doi: 10.1371/journal.pone.0236575 (PMC7386633; doi:10.1371/journal.pone.0236575)
Supplement: S6 File — Results of model selection (Table A) and model averaging approaches (Table B). (DOCX) [file pone.0236575.s006.docx]

**S6 File: Results of model selection (Table A) and model averaging approaches (Table B).**

**Table A:** The six best models selected by the model selection approach based on their AICc values (∆AICc<2).

|  | **Intercept** | **alter_**  **hydro** | **alter_**  **morpho** | **alter_**  **wq** | **density** | **dist_sea** | **max_height** | **pass_ratio** | **AICc** | **∆AICc** |
| --- | --- | --- | --- | --- | --- | --- | --- | --- | --- | --- |
| 1 | -7.05 | 0.70 |  | 0.68 | 4.99 | 0.21 | 3.72 |  | 274.81 | 0.00 |
| 2 | -6.60 | 0.72 |  | 0.69 | 4.56 | 0.22 | 3.78 | -0.78 | 274.89 | 0.08 |
| 3 | -7.37 | 0.65 | 0.38 | 0.56 | 5.36 | 0.22 | 3.76 |  | 275.21 | 0.40 |
| 4 | -6.94 | 0.67 | 0.34 | 0.59 | 4.93 | 0.23 | 3.81 | -0.71 | 275.67 | 0.86 |
| 5 | -6.70 | 0.58 | 0.52 |  | 5.03 | 0.19 | 3.57 |  | 276.08 | 1.27 |
| 6 | -6.23 | 0.60 | 0.47 |  | 4.61 | 0.20 | 3.59 | -0.67 | 276.66 | 1.85 |

**Table B:** Relative importance of the variables calculated by the model averaging approach using the six best models selected. The variables with a sum of AICc weights >0.50 were included in the final LDF model.

|  | **alter_hydro** | **density** | **dist_sea** | **max_height** | **alter_wq** | **alter_morpho** | **pass_ratio** |
| --- | --- | --- | --- | --- | --- | --- | --- |
| **Sum of AICc weights** | 1 | 1 | 1 | 1 | 0.79 | 0.55 | 0.46 |
| **Number of models containing the variable** | 6 | 6 | 6 | 6 | 4 | 4 | 3 |
