# Supplementary material for: Modeling diadromous fish loss from historical data: Identification of anthropogenic drivers and testing of mitigation scenarios
Source: PLoS One. 2020 Jul 28;15(7):e0236575. doi: 10.1371/journal.pone.0236575 (PMC7386633; doi:10.1371/journal.pone.0236575)
Supplement: S7 File — (DOCX) [file pone.0236575.s007.docx]

**S7 File: Cross-validation metrics calculated on the calibration dataset (361 sites) and on the new datasets (30% (test dataset) and 70% (training dataset) of the randomly selected calibration sites).** For the test and training datasets, the calculation was repeated 200 times; the metric values displayed correspond to the mean ± standard error.

| Dataset | MAE^1^ | RMSE^2^ | R² ^3^ |
| --- | --- | --- | --- |
| Calibration | 0.19 | 0.28 | 0.63 |
| Test (30% of sites) | 0.20 ±0.001 | 0.30±0.002 | 0.58±0.004 |
| Training (70% of sites) | 0.18 ±0.001 | 0.28±0.001 | 0.63±0.002 |

^1^ mean absolute error

^2^ root mean squared error

^3^ squared correlation between the response variable and the predicted values
